# IFI16 promotes human embryonic stem cell trilineage specification through interaction with p53

**DOI:** 10.1038/s41536-020-00104-0

**Published:** 2020-10-29

**Authors:** Qian He, Zubiao Wu, Wei Yang, Doukou Jiang, Chaofeng Hu, Xiaofei Yang, Ning Li, Furong Li

**Affiliations:** 1 Translational Medicine Collaborative Innovation Center, The Second Clinical Medical College (Shenzhen People’s Hospital), Jinan University, 518020 Shenzhen, China; 2 Integrated Chinese and Western Medicine Postdoctoral Research Station, Jinan University, 510632 Guangzhou, China; 3 Guangdong Engineering Technology Research Center of Stem Cell and Cell therapy, 518020 Shenzhen, China; 4 Shenzhen Key Laboratory of Stem Cell Research and Clinical Transformation, 518020 Shenzhen, China; 5 Present Address: Translational Medicine Collaborative Innovation Center, The Second Clinical Medical College (Shenzhen People’s Hospital), Jinan University, 1017 Dongmen North Road, 518020 Shenzhen, China

**Keywords:** Embryonic stem cells, Transcriptional regulatory elements

## Abstract

Transcriptional regulation plays an essential role in the self-renewal and differentiation of human embryonic stem cells (hESCs). However, how external signals disrupt the self-renewal regulatory network and further drive hESC differentiation remains largely unknown. Here, we found the immune regulative protein, gamma-interferon-inducible protein 16 (IFI16) was involved in the regulation of both self-renewal and differentiation gene expression during hESC trilineage specification through interaction with p53. IFI16 expression levels were upregulated through JNK activation. IFI16 knockdown delayed the downregulation of self-renewal gene expression and suppressed the upregulation of differentiation gene expression, while IFI16 overexpression accelerated trilineage specification. Furthermore, IFI16 stabilized p53-binding in the genome through IFI16-p53 interaction and differentially regulated self-renewal and differentiation gene expression. Together, our results suggest a particular role of IFI16 in differential gene expression regulation during trilineage specification of hESCs in a manner that is dependent on the genome-wide profile of p53-binding directed by IFI16-p53 interaction.

## Introduction

Although human embryonic stem cells (hESCs) and somatic cells share almost identical DNA sequence information, hESCs maintain pluripotency through selective gene expression. According to the protocol proposed by Shinya Yamanaka, somatic cells can be reprogrammed into pluripotent stem cells by ectopic expression of transcription factors such as OCT4 and SOX2^1^, suggesting that transcriptional regulation plays a key role in the pluripotency maintenance of hESCs. Indeed, OCT4, SOX2, and Nanog form a core self-renewal regulatory network to maintain the pluripotent state of hESCs^2,3^. The cooperation of these transcription factor regulators, epigenetic modifiers and effectors of external signaling pathways maintain the pluripotency of hESCs^2^. Although the transcriptional regulatory network of the pluripotent state in hESCs has been described, how external signals disrupt the core self-renewal regulatory network and further drive the differentiation of hESCs remains largely unclear.

Gamma interferon inducible protein 16 (IFI16), a member of p200 family, was first identified in lymphoid cells and was considered as an intracellular DNA sensor in the regulation of the immune responses^4–6^. Beyond that, evidence supports that IFI16 contributes to genome remodeling in virus defense through interaction with histone H2B or H3K9 methyltransferase^7,8^. Moreover, an observation that IFI16 upregulation induced by LIF mediating the cell cycle arrest has been demonstrated in medullary thyroid carcinoma cells^9^. Therefore, those findings above demonstrate a potential role of IFI16 in cell proliferation and nuclear protein binding. However, in addition to immunoregulation, whether IFI16 participates in other physiological process especially in self-renewal and differentiation of hESCs is still unknown.

As one of the most important tumor suppressors, p53 functions in cell cycle arrest and apoptosis during aberrant oncogene activation and senescence of somatic cells after genomic instability^10,11^. Beyond that, p53 also plays an important role in the self-renewal and differentiation of ESCs^12^. p53 expression level has been upregulated during hESCs early differentiation and p53 knockdown reduces the spontaneous differentiation^13^. In addition, p53 suppresses Nanog expression and sufficiently induces mouse embryonic stem cells (mESCs) differentiation^14^, and OCT4 maintains the pluripotency by inactivating p53 in hESCs^15^. Furthermore, p53 regulates LncPRESS1 and coordinates Wnt/Nodal signals contributing to hESCs differentiation^16,17^. However, activation of a transcription factor would result in a spectrum of gene expression change and further affect the downstream signals^18,19^. Despite an extensive understanding of p53 function in regulation of the self-renewal and differentiation of ESCs, the genome-wide profiling of p53 targets and corresponding gene expression during hESCs differentiation is still largely unknown. Remarkably, the crystal structures of both HIN-A and HIN-B domains of IFI16 interact with p53^20^. Therefore, it is promising to identify the IFI16/p53 interaction endogenously and explore whether IFI16/p53 signal could directly affect hESCs differentiation.

In this study, we aimed to investigate whether IFI16 would participate in the regulation of hESCs differentiation. We report that JNK activation induced IFI16 upregulation which stabilized p53-binding in the genome through IFI16-p53 interaction and further differentially regulated self-renewal and differentiation gene expression to determine the trilineage specification of hESCs.

## Results

### IFI16 is specifically upregulated during trilineage specification

**Fig. 1:**
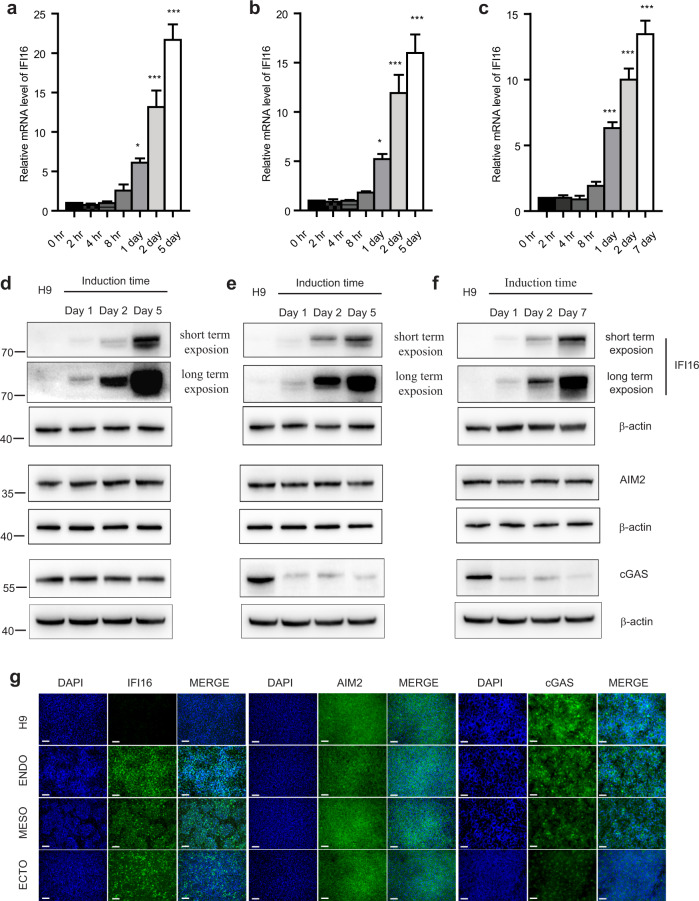
Upregulation of IFI16 expression during trilineage specification. **a**–**c** Quantitative PCR analysis of IFI16 mRNA levels in H9 cells and differentiated trilineage (**a**, endoderm; **b**, mesoderm; **c**, ectoderm; *n* = 4 in each group) for indicated periods of time. The relative mRNA levels of IFI16 in indicated time courses were calculated relatively to which at 0 h. **d**–**f** Representative immunoblots of IFI16, AIM2, and cGAS from H9 cells and differentiated trilineage (**d**, endoderm; **e**, mesoderm; **f**, ectoderm) for indicated periods of time. β-actin serves as a loading control. **g** Representative immunofluorescence images staining with antibodies against IFI16, AIM2, and cGAS in H9 cells and the differentiated trilineage. DAPI serves as a nucleus indicator. Scale bar, 200 µM. ENDO, endoderm; MESO, mesoderm; ECTO, ectoderm. All data were presented as mean ± SEM. Comparisons between groups for statistical significance were performed with one-way ANOVA with Tukey’s post hoc test. **P < 0.01, ***P < 0.001 versus H9.

To investigate the role of gamma-interferon-inducible protein 16 (IFI16) in the self-renewal and differentiation of human embryonic stem cells (hESCs), we used hESC line H9 to derive embryonic stages of endoderm, mesoderm and ectoderm. Initially we measure the IFI16 expression levels in hESCs and trilineage differentiated cells, and found that the IFI16 mRNA and protein levels were gradually upregulated during the trilineage specification. As shown in Fig. 1a–f and Supplementary Fig. 1d–f, a low expression level of IFI16 was observed in H9 cells, while IFI16 expression reached a medium level at Day 2 of trilineage specification and had a huge potentiation in the final stages (Day 5 of endoderm/mesoderm and Day 7 of ectoderm). To verify the specificity of IFI16 regulation during trilineage specification, we examined the expression levels of another two intracellular DNA sensor, Absent In Melanoma 2 (AIM2) and cyclic GMP-AMP Synthase (cGAS). In contrast, the mRNA and protein expression levels of AIM2 were not significantly changed during the trilineage specification (Fig. 1d–f and Supplementary Fig. 1a–f). In addition, the expression level of cGAS was not changed during the endoderm specification, while remarkably downregulated when differentiating into the mesoderm and ectoderm (Fig. 1d–f and Supplementary Fig. 1a–c and g–i). To confirm the IFI16 expression during the trilineage specification, the IFI16 protein were in situ stained through immunofluorescence. Consistent with the results from western blots, low IFI16 expression was evident in H9 cells, while IFI16 expression levels were greatly upregulated in the nuclei of endoderm, mesoderm and ectoderm cells (Fig. 1g). Meanwhile, AIM2 had a relatively stable expression during the trilineage specification (Fig. 1g), and the expression level of cGAS was reduced during the mesoderm and ectoderm specification, while no significant change was observed during the endoderm specification (Fig. 1g). Together, these results provide the initial evidence that IFI16 protein levels are specifically upregulated during the trilineage specification.

### JNK activation mediates IFI16 upregulation during trilineage specification

**Fig. 2:**
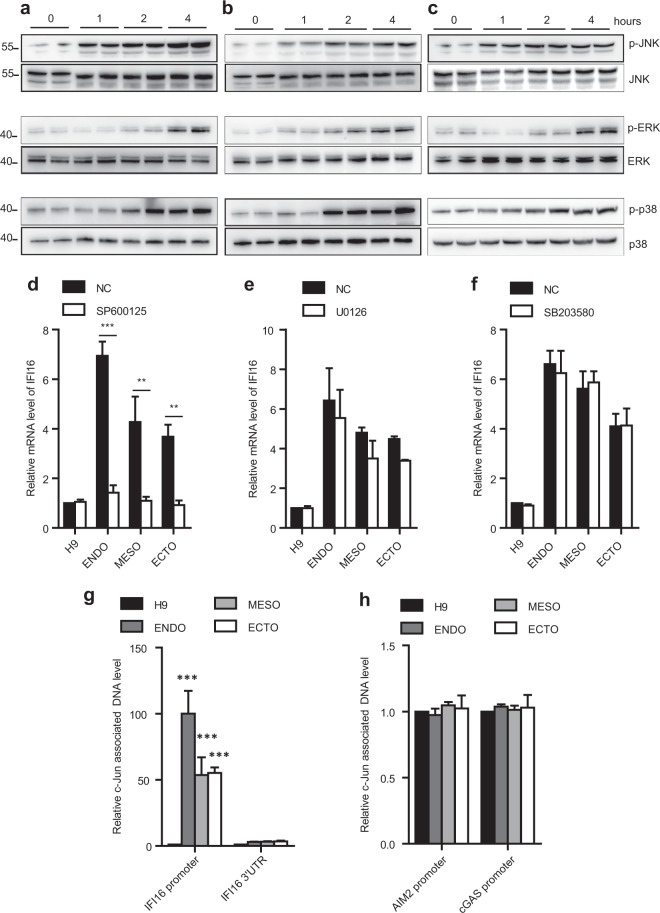
JNK activation is responsible for IFI16 upregulation during trilineage specification. **a**–**c** Representative immunoblots of total lysates from H9 cells and differentiated trilineage (**a**, endoderm; **b**, mesoderm; **c**, ectoderm) for indicated periods of time and probed with the antibodies for p-JNK, JNK, p-ERK, ERK, for p-p38 and p38. **d**–**f**, quantitative PCR analysis of IFI16 mRNA levels in H9 cells and differentiated trilineage after incubation with SP600125 (10 µM, *n* = 4, (**d**), U0126 (10 µM, *n* = 3, (**e**), and SB203580 (10 µM, *n* = 4, **f** The relative mRNA level of IFI16 was calculated relatively to which in H9 cells. **g**, **h** quantitative PCR examination of IFI16 promoter (*n* = 4), IFI16 3’UTR (*n* = 4), AIM2 promoter (*n* = 3), and cGAS promoter (*n* = 3) levels pulled-down by c-Jun antibodies in H9 cells and differentiated trilineage. The relative c-Jun associated DNA level was calculated relatively to which in H9 cells. ENDO, endoderm; MESO, mesoderm; ECTO, ectoderm. NC, negative control. All data were presented as mean ± SEM. Comparisons between groups for statistical significance were performed with one-way ANOVA with Tukey’s post hoc test (**g**, **h**) or two-way ANOVA with Bonferroni post hoc test (**d**–**f**). *P < 0.05, **P < 0.01, ***P < 0.001 versus 0 h, NC or H9.

We next explored the possible mechanism underlying the upregulation of IFI16 expression. The observation from quantitative real-time PCR (qPCR) results (Fig. 1a–c) suggested that the upregulation of IFI16 expression was probably attributed to its transcriptional activation. Given that the mitogen-activated protein kinase (MAPK) plays an important role in the regulation of cellular proliferation and differentiation^21–23^, we assessed the activities of MAPK families including extracellular signal-regulated kinases (ERKs, p42, and p44), the c-Jun N-terminal kinases (JNKs) and the p38 MAP kinases. As shown in Fig. 2a–c, the ERK, JNK, and p38 kinases were all activated within 4 h after the trilineage specification as evidenced by their respective phosphorylations. To investigate the role of MAPKs in the upregulation of IFI16, we further examined the IFI16 mRNA levels in the trilineage cells after inhibition of these kinases. Incubating H9 cells with SP600125, U0126, or SB203580, the specific inhibitors of JNK, ERK, or p38 MAP kinases respectively, did not change the IFI16 mRNA levels (Fig. 2d–f). However, the upregulation of IFI16 during the trilineage specification was greatly inhibited by SP600125 (Fig. 2d). In contrast, no significant changes were observed in IFI16 mRNA expression after U0126 or SB203580 incubation (Fig. 2e, f). Since c-Jun has a validated binding motif in IFI16 promoter^24^, we then performed chromosome immunoprecipitation (ChIP) to evaluate the transcriptional activity of IFI16 promoter. We found that the IFI16 promoter was remarkably enriched during the trilineage specification by c-Jun antibodies, while the binding levels of IFI16 3’ untranslated regions (UTR), AIM2 and cGAS promoter were not affected (Fig. 2g, h). Taken together, these results suggest that JNK pathway specifically participates in the transcriptional activation of IFI16.

### IFI16 knockdown inhibits trilineage specification

**Fig. 3:**
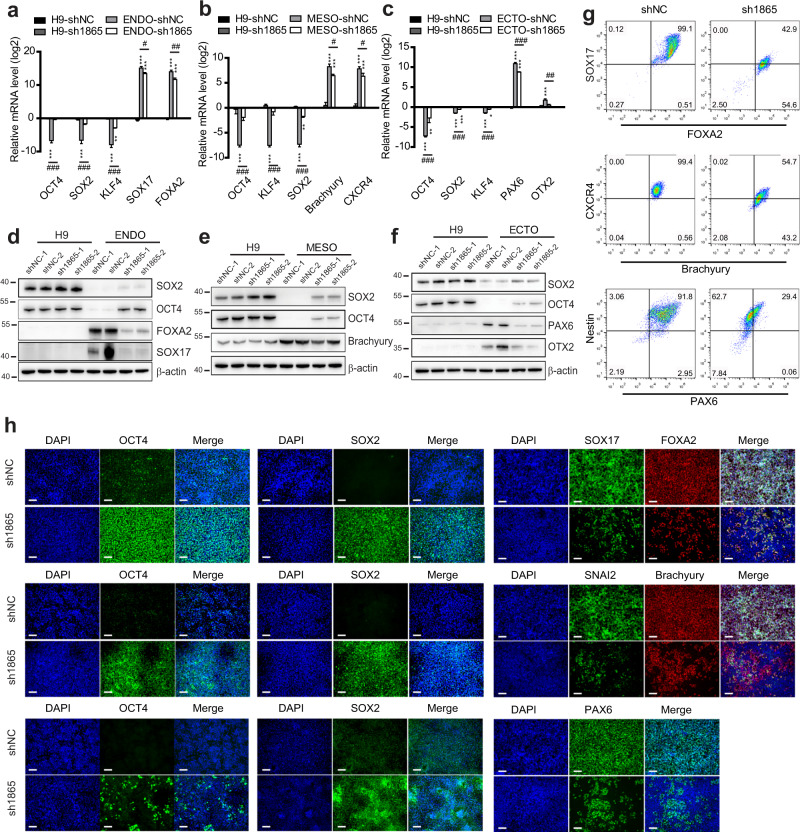
IFI16 knockdown by sh1865 inhibits trilineage specification. **a**–**c** Quantitative PCR examination of OCT4, SOX2, KLF4, SOX17, FOXA2, CXCR4, Brachyury, PAX6, and OTX2 mRNA levels in H9 cells and differentiated trilineage (**a**, endoderm; **b**, mesoderm; **c**, ectoderm; *n* = 4 in each group) infected with sh1865 or shNC. The relative mRNA level of each gene was calculated relatively to which in H9-shNC group. **d–f** Representative immunoblots of total lysates from H9 cells and differentiated trilineage (**d**, endoderm; **e**, mesoderm; **f**, ectoderm) infected with sh1865 or shNC and probed with the antibodies for OCT4, SOX2, SOX17, FOXA2, Brachyury, PAX6, and OTX2. β-actin serves as a loading control. **g** Flow cytometric analysis of SOX17^+^/FOXA2^+^, Brachyury^+^/CXCR4^+^, and PAX6^+^/Nestin^+^ population in differentiated trilineage infected with sh1865 or shNC, The signals in the fourth quadrant indicate endoderm, mesoderm, or ectoderm population. The number in each quadrant means the proportion in total cell population. **h** Representative immunofluorescence images staining with antibodies against OCT4, SOX2, SOX17, FOXA2, Brachyury, SNAI2, and PAX6 in the differentiated trilineage infected with sh1865 or shNC. Upper rows, endoderm; middle rows, mesoderm; bottom rows, ectoderm. DAPI serves as a nucleus indicator. Scale bar, 200 µM. ENDO, endoderm; MESO, mesoderm; ECTO, ectoderm. NC, negative control. All data were presented as mean ± SEM. Comparisons between groups for statistical significance were performed with one-way ANOVA with Tukey’s post hoc test (**a**–**c**). *P < 0.05, **P < 0.01, ***P < 0.001 versus H9-NC. ^#^*P* < 0.05, ^##^*P* < 0.01, ^###^*P* < 0.001 versus H9-ENDO, H9-MESO, or H9-ECTO.

To further investigate the role of IFI16 in the self-renewal and differentiation of hESCs, we knocked down IFI16 by engineering the lentivirus in which expression of two short hairpin RNAs (shRNAs), sh1865 or sh2153. Due to the relatively low expression, sh1865 or sh2153 did not significantly change IFI16 expression in H9 cells (Supplementary Fig. 2a–d). However, IFI16 protein levels after the trilineage specification were greatly reduced by sh1865 and sh2153 (Supplementary Fig. 2a–d). Meanwhile, IFI16 knockdown did not affect the cell viability measured by the CCK-8 assays in hESCs or during the trilineage specification (Supplementary Fig. 2e–h). Notably, by qPCR and western blot analysis, we found that sh1865 and sh2153 remarkably suppressed the downregulation of the self-renewal transcriptional factors including OCT4, SOX2, and KLF4 during the trilineage specification (Fig. 3a–f, Supplementary Fig. 3a–c, Supplementary Fig. 4a–f and Supplementary Fig. 5a–c). Moreover, the upregulation of trilineage marker genes including SOX17^25^, FOXA2^26^ and CXCR4^27^ for endoderm, Brachyury^28^ and CXCR4^29^ for mesoderm, and PAX6^30^, OTX2^31^ for ectoderm, were significantly reduced by sh1865 and sh2153 (Fig. 3a-f, Supplementary Fig. 3a–c, Supplementary Fig. 4a–f and Supplementary Fig. 5a–c).

To further validate the role IFI16 in regulation of the self-renewal and differentiation gene expression, we performed flow cytometry and immunofluorescence. The SOX17^+^/FOXA2^+^ population was referred to as endoderm cells and Brachyury^+^/CXCR4^+^, PAX6^+^/Nestin^+^^32^ were referred to as mesoderm and ectoderm cells, respectively. The endoderm, mesoderm and ectoderm population were all remarkably decreased by sh1865 and sh2153 (Fig. 3g, Supplementary Fig. 3d–f, Supplementary Fig. 4g and Supplementary Fig. 5d–f). Moreover, the fluorescence intensity of OCT4 and SOX2 was significantly upregulated by sh1865 and sh2153 during the trilineage specification, while trilineage marker genes including SOX17, FOXA2, Brachyury, SNAI2^33^ and PAX6 were significantly reduced by sh1865 and sh2153 (Fig. 3h, Supplementary Fig. 3g–i, Supplementary Fig. 4h and Supplementary Fig. 5g–i). Collectively, our results suggest that IFI16 knockdown delays the downregulation of self-renewal gene expression and suppresses the upregulation of differentiation gene expression.

### IFI16 overexpression accelerates trilineage specification

To provide further evidence that regulation of hESCs self-renewal and differentiation by IFI16, we generated a lentiviral construct encoding the IFI16 gene that can be induced by doxycycline (DOX). As shown in Supplementary Fig. 6a, b, after infection, DOX induced a time course dependent exogenesis expression of IFI16 in H9 cells. Notably, DOX-induced IFI16 expression did not significantly change OCT4 and SOX2 protein levels in H9 cells (Supplementary Fig. 6c, d). In addition, alkaline phosphatase (AP) staining showed that there was no significant difference among the AP positive proliferative colonies in DOX-induced IFI16 expression group, no-DOX induction group and negative control group (Supplementary Fig. 6e). These results indicate that IFI16 overexpression alone does not affect the self-renewal ability of the hESCs.

**Fig. 4:**
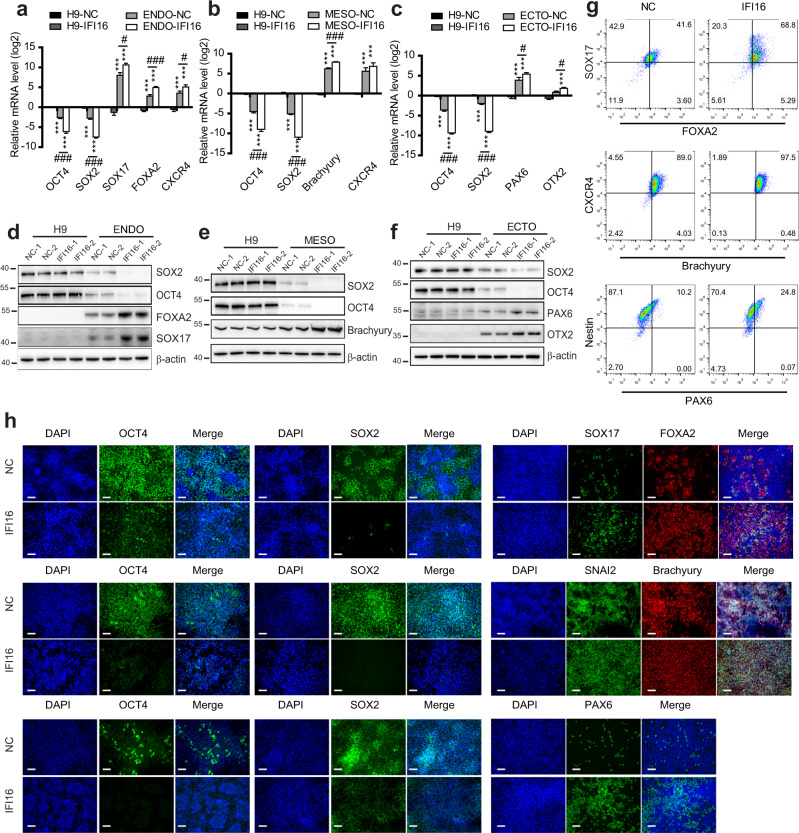
IFI16 overexpression accelerates trilineage specification. **a**–**c** Quantitative PCR analysis of OCT4, SOX2, SOX17, FOXA2, CXCR4, Brachyury, PAX6, and OTX2 mRNA levels in H9 cells and differentiated trilineage on Day 2 (**a**, endoderm; **b**, mesoderm; **c**, ectoderm; *n* = 4 in each group) infected with IFI16 or NC. The relative mRNA level of each gene was calculated relatively to which in H9-NC group. **d**–**f** Representative immunoblots of total lysates from H9 cells and differentiated trilineage on Day 2 (**d**, endoderm; **e**, mesoderm; **f**, ectoderm) infected with IFI16 or NC and probed with the antibodies for OCT4, SOX2, SOX17, FOXA2, Brachyury, PAX6, and OTX2. β-actin serves as a loading control. **g** Flow cytometric analysis of SOX17^+^/FOXA2^+^, Brachyury^+^/CXCR4^+^, and PAX6^+^/Nestin^+^ population in differentiated trilineage on Day 2 infected with IFI16 or NC, The signals in the fourth quadrant indicate endoderm, mesoderm, or ectoderm population. The number in each quadrant means the proportion in total cell population. **h** Representative immunofluorescence images staining with antibodies against OCT4, SOX2, SOX17, FOXA2, Brachyury, SNAI2, and PAX6 in the differentiated trilineage on Day 2 infected with IFI16 or NC. Upper rows, endoderm; middle rows, mesoderm; bottom rows, ectoderm. DAPI serves as a nucleus indicator. Scale bar, 200 µM. ENDO, endoderm; MESO, mesoderm; ECTO, ectoderm. NC, negative control. All data were presented as mean ± SEM. Comparisons between groups for statistical significance were performed with one-way ANOVA with Tukey’s post hoc test (**a**–**c**). ***P < 0.001 versus H9-NC. ^#^*P* < 0.05, ^###^*P* < 0.001 versus H9-ENDO, H9-MESO, or H9-ECTO.

In the next attempt to study whether IFI16 overexpression regulates hESCs differentiation, we measured the differentiation gene expression. Results from western blots showed that the expression of trilineage marker genes including SOX17, FOXA2, Brachyury, SNAI2, and PAX6 were not affected by DOX-induced IFI16 expression in the final stages of trilineage induction (Supplementary Fig. 7a–f). Moreover, as shown in Supplementary Fig. 7g–i, the endoderm, mesoderm, and ectoderm population in FACS were not changed either. Due to the relatively high expression levels of IFI16 in the final stages of trilineage induction (Fig. 1), we thereafter hypothesized that IFI16 overexpression would be involved in the regulation of the hESCs self-renewal and differentiation in the early stage of trilineage induction. Indeed, on Day 2 of trilineage induction, the downregulation of OCT4 and SOX2 levels were further enhanced by IFI16 expression according to the qPCR and western blot analysis (Fig. 4a-f and Supplementary Fig. 8a–c). In addition, the upregulation of trilineage marker genes including SOX17, FOXA2, Brachyury, OTX2, and PAX6 were also potentiated (Fig. 4a-f and Supplementary Fig. 8a–c). Moreover, the endoderm and ectoderm population in FACS were greatly enlarged by IFI16 expression, while the mesoderm population had a moderate change (Fig. 4g and Supplementary Fig. 8d–f). Furthermore, the fluorescence intensity of OCT4 and SOX2 was significantly downregulated after IFI16 expression (Fig. 4h and Supplementary Fig. 8g–i). In the meanwhile, SOX17, FOXA2, Brachyury, SNAI2, and PAX6 were significantly upregulated (Fig. 4h and Supplementary Fig. 8g–i). Taken together, the results above suggest that IFI16 overexpression accelerates the downregulation of self-renewal gene expression and upregulation of the differentiation gene expression in the early stage of trilineage induction.

### IFI16 interacts with p53 during trilineage specification

**Fig. 5:**
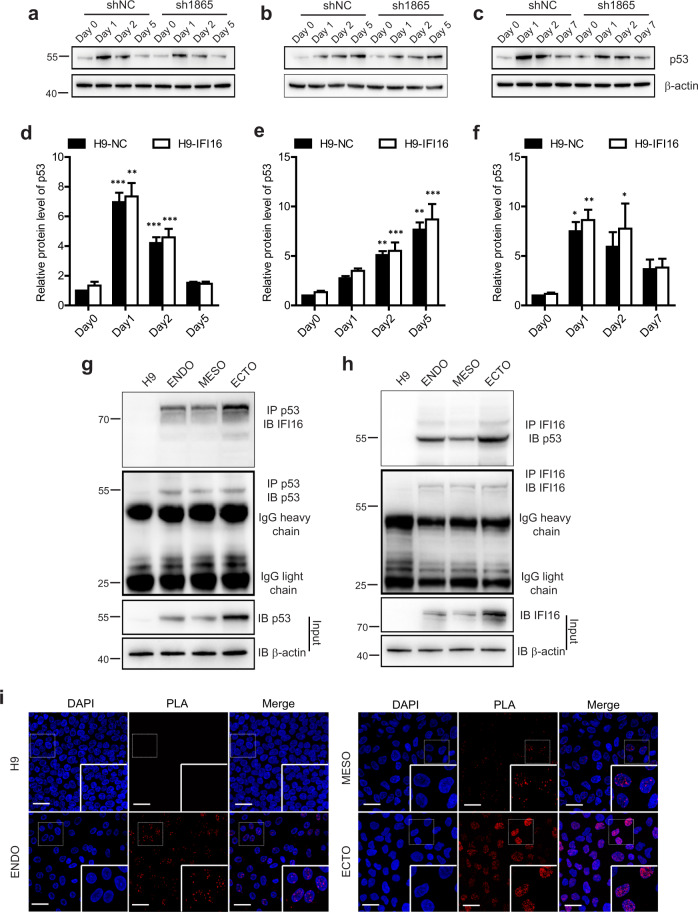
IFI16 interacts with p53 during trilineage specification. **a**–**c** Representative immunoblots of p53 from H9 cells and differentiated trilineage (**a**, endoderm; **b**, mesoderm; **c**, ectoderm) infected with sh1865 or shNC. **d**–**f** Statistics for **a**–**c** respectively, *n* = 4 for each group. The relative protein level of p53 was calculated relatively to which in H9-shNC group. **g**–**h** lysates of H9 cells and differentiated trilineage on Day 2 precipitated with p53 (**g**) and IFI16 (**h**) antibodies, and immunoblotted with indicated antibodies. β-actin serves as a loading control. **i** PLA analysis of IFI16-p53 interaction in H9 cells and differentiated trilineage on Day 2. DAPI serves as a nucleus indicator. Dashed boxes indicate the zoom in reagions in each image. Scale bar, 50 µM. ENDO, endoderm; MESO, mesoderm; ECTO, ectoderm. NC, negative control. All data were presented as mean ± SEM. Comparisons between groups for statistical significance were performed with two-way ANOVA with Bonferroni post hoc test (**d**–**f**). *P < 0.05, **P < 0.01, ***P < 0.001 versus H9-NC Day 0.

The results above suggest the important role of IFI16 in the regulation of hESCs self-renewal and differentiation. To investigate the possible mechanism underlying this regulation, we assessed p53 expression since p53 has been considered as the downstream signal of IFI16. As shown in Fig. 5a–f, p53 had a relatively low expression level in H9 cells and was remarkably increased during trilineage specification, though the pattern of its upregulation was different in different germ layers. Notably, IFI16 knockdown did not significantly change the pattern of p53 expression during trilineage specification (Fig. 5a–f), indicating that the IFI16 role in the regulation of hESCs self-renewal and differentiation was not attributed to p53 expression. Since p53 could interact with IFI16 and mediated IFI16 function^20^, we were thereafter encouraged to verify the IFI16-p53 interaction during trilineage specification and investigate whether this interaction contributed to hESCs self-renewal and differentiation. To ensure relatively high expression levels of p53 and IFI16, we performed co-immunoprecipitation (co-IP) on Day 2 of trilineage induction. Indeed, solid interaction between p53 and IFI16 was observed during trilineage specification (Fig. 5g, h). On the contrary, rare IFI16-p53 interaction was found in H9 cells due to its relatively low levels of p53 and IFI16 (Fig. 5g, h). Consistently, results from proximity ligation assay (PLA) also demonstrated that IFI16 could interact with p53 in the nuclei of trilineage cells in situ, while the fluorescence signals were not found in H9 cells (Fig. 5i). Together, these results provide the evidence that IFI16 interacts with p53 during trilineage specification.

### IFI16 facilitates p53 binding in the genome

**Fig. 6:**
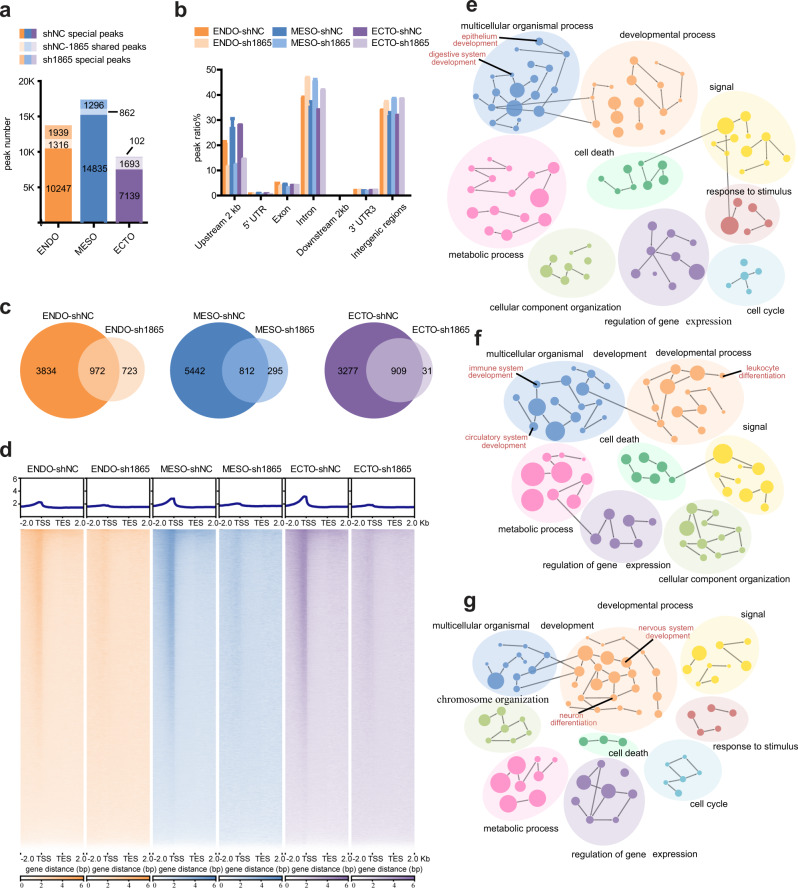
Genomic profiling of p53 peaks after IFI16 knockdown during trilineage specification. **a** Histogram of p53 peak number after IFI16 knockdown during trilineage specification. **b** The distribution ratio of p53 peaks after IFI16 knockdown during trilineage specification. **c**, **d** Venn diagrams (**c**) and heatmaps (**d**) of genes enriched by p53 peaks after IFI16 knockdown during trilineage specification. **e**–**g** Biological processes gene ontology (GO) analysis for genes enriched by p53-shNC special peaks in differentiated trilineage (**e**, endoderm; **f**, mesoderm; **g**, ectoderm). Each node represented a specific GO term, and the node size indicated the number of genes in the GO term. The GO term annotations are listed in the Supplementary Dataset 1. ENDO, endoderm; MESO, mesoderm; ECTO, ectoderm.

To determine the roles of IFI16-p53 interaction during trilineage specification, we performed ChIP-seq to derive a genome-wide profile of p53-binding genes after IFI16 knockdown by sh1865. p53 binding sites were mapped on Day 2 of trilineage induction, when IFI16-p53 interaction was observed. After peak-calling, 11563, 15697, and 8832 p53 peaks were identified in endoderm, mesoderm, and ectoderm lineage induction of shNC group, while 3255, 2158, and 1795 p53 peaks were obtained, respectively, in sh1865 group (Fig. 6a). And the 10247, 14835, and 7139 special peaks in each lineage of shNC group were considered as IFI16 regulated p53 peaks (Fig. 6a). These results provided the initial evidence that IFI16 knockdown reduces p53-binding in the genome during trilineage specification. To validate whether the identified peaks contain p53 response elements, de novo motif analysis was conducted using MEME. We compared the sequences from the highly enriched peaks with the p53 binding motif sequences in the JASPAR database and found these sequences were enriched with p53 binding motifs (Supplementary Fig. 9a).

In addition, consistent with the findings from previous studies, the majority of p53-binding sites in the differentiated trilineage genome were localized in introns^34^. Other p53-binding sites are mainly scattered in the intergenic regions (>2 kb upstream/downstream of the transcription start sites), within 2 kb upstream of transcriptional start site (TSS), within 2 kb downstream of transcriptional end site (TES) and exons. No evident differences were observed in the distribution of p53-binding sites among different lineage differentiation (Fig. 6b). However, IFI16 knockdown remarkably reduced the distribution ratio of p53 peaks within 2 kb upstream of TSS (Fig. 6b), which indicated that IFI16 mainly stabilizes the p53-binding pattern in the promoter regions during the different lineage differentiation of hESCs.

To investigate the biological significance of the IFI16 regulated p53 peaks during trilineage specification, we referred these p53 peaks to specific RefSeq genes if the peaks were within 2 kb of the genes, and identified 4806, 6245, and 4186 genes with p53 peaks in the endoderm, mesoderm, and ectoderm lineage, respectively (Fig. 6c, d). Consistently, the numbers of the p53-binding genes dropped to 1695, 1107, and 940 after IFI16 knockdown (Fig. 6c, d), and we defined the 3834, 5442, and 3277 genes associated special peaks in shNC groups as IFI16-p53 regulated genes. By using Cytoscape analysis for biological processes gene ontology (GO) terms, the biological functions of these p53-binding genes were further annotated. As expected, the genes involved in biological processes associated with p53 function, for example, “cell death” and “cell cycle” were enriched in the differentiated trilineages of shNC (Supplementary Fig. 9b–d). In addition, we found significant enrichment for genes contributes to development including “anatomical structure development” and “developmental process” (Supplementary Fig. 9b–d). Notably, “signal” and “regulation of gene expression” related genes were also observed in IFI16-p53 regulated gene group of differentiated trilineages (Fig. 6e–g). Moreover, “digestive tract development” and tepithelium development” was specially enriched for endoderm, “immune system development” and ecirculatory system development” for mesoderm and “nervous system development” and us systedifferentiation” for ectoderm (Fig. 6e–g). Together, the results above point to a possibility that IFI16 stabilizes p53-binding in the genome which probably contributes to trilineage specification of hESCs.

### IFI16-p53 interaction differentially regulates of self-renewal and differentiation gene expression

**Fig. 7:**
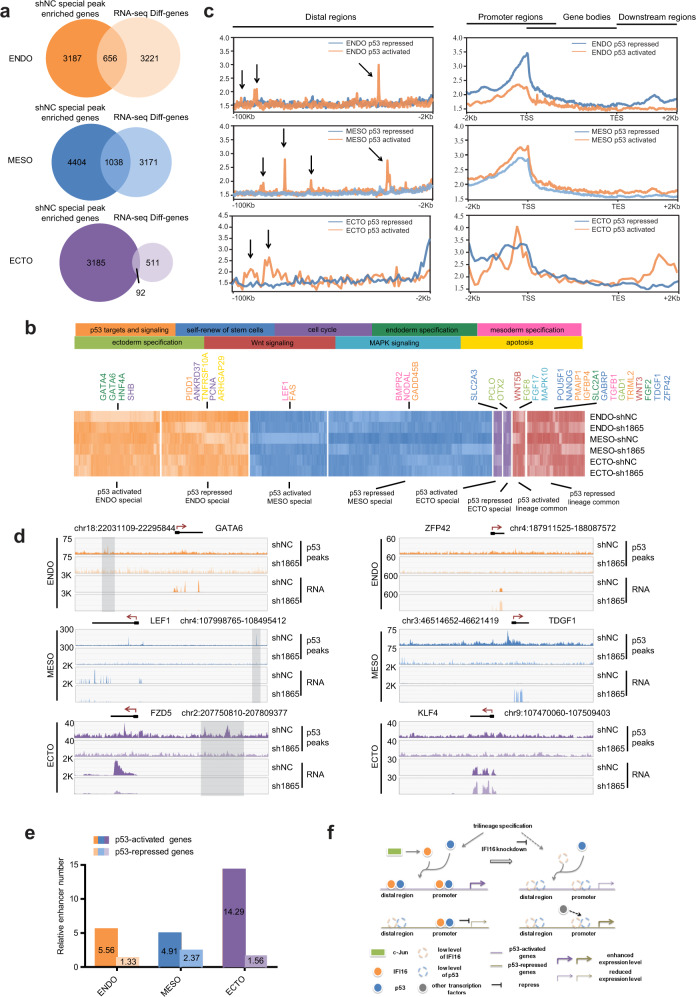
Differentially regulating self-renewal and differentiation gene expression by p53-shNC special peaks. **a** Venn diagrams of p53-shNC special peak enriched genes and genes differentially expressed after IFI16 knockdown during trilineage specification. **b** Heatmaps (bottom) of genes specially and commonly regulated by p53-shNC special peaks after IFI16 knockdown and corresponding function/pathway (up) during trilineage specification. **c** Averaged p53 peaks in distal, promoter and gene body, and proximal downstream regions of p53-activated and p53-repressed genes in differentiated trilineage. The arrows indicate the averaged p53 peaks in distal regions of p53-activated genes. **d** Representative genomic views of p53 peaks and RNA levels at p53-activated (left) and p53-repressed (right) genes after IFI16 knockdown in differentiated trilineage. The shadow boxes indicate significant p53 peaks in the shNC group. **e** Histogram of the relative numbers of enhancer overlapped with p53 peaks in the TSS upstream (2 to 100 kb) of p53-activated and p53-repressed genes in differentiated trilineage. **f** Schematic diagram depicting a working model for IFI16-p53 interaction differentially regulating gene expression during trilineage specification. ENDO, endoderm; MESO, mesoderm; ECTO, ectoderm.

We then explored whether the IFI16 regulated p53 peaks would contribute to the transcription regulation of the nearby genes. We performed RNA-seq and identified 3877, 4209, and 603 differential genes, respectively, in the endoderm, mesoderm, and ectoderm lineage after IFI16 knockdown (Fig. 7a). Moreover, significant enrichment for genes contributes to development including “anatomical structure development”, “regulation of developmental process” and “animal organ development” were observed in these differential genes using Cytoscape analysis (Supplementary Fig. 10a–c), which is consistent with the finding above that IFI16 knockdown inhibits trilineage specification. After integrating the IFI16 regulated p53 peaks with differential gene expression results, 656, 1038, and 92 genes were determined to be p53 targeting genes (Fig. 7a), which have one p53-binding peak at least and RNA level changed in the endoderm, mesoderm, and ectoderm lineage after IFI16 knockdown.

Notably, besides correlating with the upregulation of genes (termed p53 activated genes), binding of p53 probably also represses gene expression (termed p53 repressed genes) which include some key transcription regulators in hESCs (Fig. 7b and Supplementary Fig. 10d). The expression of FGF8 was reduced associated with decreased p53 peaks during trilineage specification (Fig. 7b). Moreover, the expression of endoderm marker genes, for example, GATA4, GATA6, and HNF4A were specifically downregulated in the endoderm lineage after IFI16 knockdown (Fig. 7b). Meanwhile, the expression of mesoderm marker gene LEF1 and ectoderm marker gene PCLO, OTX2 also decreased in the mesoderm and ectoderm lineage, respectively (Fig. 7b), correlated with the reduced p53 peaks. Especially, the expression levels of OCT4, NANOG, KLF4, and ZFP42 were upregulated when p53 peaks decreased (Fig. 7b), indicating that IFI16-p53 would inhibit self-renewal gene expression. In addition, genes related to Wnt signaling, MAPK signaling, apoptosis and p53 signaling were also regulated by IFI16-p53 interaction (Fig. 7b).

To further investigate the possible mechanism underlying the differential gene transcriptional outcome, i.e., activation or repression regulated by p53, we compared the p53 binding patterns within the p53 activated and repressed genes. As shown in Fig. 7c, we evaluated the p53 peaks in gene body, distal (2–100 kb upstream of the TSS), promoter-proximal (within 2 kb upstream of TSS) and proximal downstream (within 2 kb downstream of TES) regions and did not found an obvious difference in p53 peaks of gene body regions between p53 activated and repressed genes. Meanwhile, the differential binding of the p53 in the promoter and proximal downstream regions was not consistent among the trilineages (Fig. 7c, d). However, more p53 peaks were found in the distal regions of p53 activated genes (Fig. 7c, d), which indicating that the binding of p53 in the distal upstream region would contribute to the activation of gene expression. Since enhancers are usually located in the distal upstream region of genes and facilitated the transcription activation, the alignment of p53 peaks in distal upstream region (2 to 100 kb) of p53-activated and p53-repressed genes with the validated enhancer sequences in the FANTOM5 were performed to identify the potential p53 binding enhancers. As shown in Fig. 7e, p53 peaks in the distal upstream region of p53-activated genes greatly enriched more overlapped enhancers, compared with p53-repressed genes, which suggested that p53 binding enhancers in the distal upstream region determine the transcription fate of p53 binding genes. Collectively, all these results are consistent with an explanation that the upregulated expression of IFI16, which interacts with p53, would facilitate p53-binding in the genome to contribute to differentially regulation of self-renewal and differentiation gene expression and further determine the trilineage specification of hESCs (Fig. 7f).

## Discussion

Here, we report a novel mechanism by which IFI16-p53 interaction promotes hESCs differentiating into trilineage cells. Several lines of evidence support this conclusion. Firstly, IFI16 expression levels were upregulated through JNK activation during trilineage specification. Secondly, IFI16 knockdown delays the downregulation of self-renewal gene expression, such as OCT4, SOX2, and KLF4 and suppresses the upregulation of differentiation gene expression including SOX17, FOXA2, CXCR4, Brachyury, PAX6, and OTX2. Thirdly, IFI16 overexpression accelerates trilineage specification through downregulation of self-renewal gene expression and upregulation the of differentiation gene expression. Lastly, IFI16 stabilizes p53-binding in the genome which correlated with the self-renewal and differentiation gene expression during hESCs trilineage specification. Together, our results suggest a particular role of IFI16 in differential gene expression regulation which is dependent on the genome-wide profile of p53-binding facilitated by IFI16-p53 interaction. Therefore, IFI16 could be a potential target to interfere in both the self-renewal and differentiation regulatory network of hESCs.

It has been mentioned above that the transcriptional regulatory network of hESCs was well studied. However, how differentiation signals disrupt the self-renewal network and further determine trilineage fate of hESCs remains largely unknown. An important implication of the current findings is that a key transcriptional factor globally regulates both self-renewal and differentiation genes contributing to the trilineage fate determination of hESCs. Here, JNK activation induces the upregulation of IFI16 expression, which leads to the enhancement of IFI16-p53 interaction. Furthermore, this interaction is suggested to activate some differentiation genes including GATA4, GATA6, LEF1, BMPR2, PCLO, and GAD1 and inhibit some self-renewal genes, such as OCT4, NANOG, KLF4, and ZFP42. Therefore, it indicates that transcriptional factors with global binding sites like p53 would regulate the expression of numerous genes and probably play crucial roles in both the self-renewal and differentiation regulatory network of hESCs.

The large number of transcriptional regulatory regions throughout the human genome dynamically interacts to control the expression of millions of base pairs^35,36^. Particularly, the expression of specific genes in different cell types is usually regulated by enhancer and promoter interaction^37,38^. Transcription factors and RNA polymerase II bind to both enhancer and promoter to mediate the interactions and control the gene transcription^38^. Here, one of the important findings of this study is that the p53-binding in some validated enhancers correlated with the activation of gene expression. Since p53 functions as a tetramer^39^, it points to a possibility that p53 binds to both enhancer and promoter of the genes and sequentially mediates enhancer-promoter loops to drive the transcriptional activation. Therefore, examination of the role of p53 in enhancer-promoter interaction formation using chromatin interaction analysis using paired-end tag sequencing (ChIA-PET)^40^ or in situ Hi–C followed by chromatin immunoprecipitation (HiChIP)^41^ would further expand our understanding of the mechanism underlying the differential regulation gene expression by IFI16-p53 interaction during hESCs trilineage specification.

IFI16 has important roles in diverse biological processes, including intracellular DNA sensing, antiviral restriction, and cell cycle regulation, while little is known about its role of differentiation regulation in hESCs. As the DNA binding of IFI16 was not dependent on the sequence specificity, specific gene regulation by IFI16 largely was relied on its interaction with other proteins containing specific DNA binding motifs^7,42,43^. A novel finding of our study is that IFI16 interacts with p53 and stabilizes p53-binding in the genome to differentially control gene expression. Notably, IFI16 expression levels were continually potentiated during hESCs trilineage specification. In the meanwhile, p53 expression levels were differentially regulated in the different lineage. Therefore, it is likely that other proteins in trilineage, especially in endoderm and ectoderm, also interacts with IFI16 and play its role in cell differentiation. Indeed, IFI16 also has a relatively high expression level in the skin, urinary bladder, and nasopharynx (https://www.proteinatlas.org/). Identifying other IFI16-interacting protein would provide us new insights into the physiological roles of IFI16 in embryogenesis or even somatic cells.

Collectively, our results point to a previously unknown role of IFI16-p53 interaction in regulating both self-renewal and differentiation gene expression and uncover the genome-wide profiling of p53 targets and corresponding gene expression during hESCs differentiation, which expands our understanding of the fate determination of hESCs.

## Methods

### hESCs culture and trilineage specification

Human embryonic stem cell (hESC) line H9 (order number: 18-1-1522) was from Cell Bank of the Shanghai Institutes for Biological Sciences of the Chinese Academy of Sciences and authenticated using Short Tandem Repeat (STR) analysis (GENETIC TESTING BIOTECHNOLOGY Co., Ltd.). hESCs were maintained in feeder-free cell culture medium mTeSR™1(STEMCELL Technologies, #85850). hESCs were passaged every 5–6 days using ReLeSR™ (STEMCELL Technologies, #05873) and had maintained a stable karyotype even until the 50th generation (Beijing Cellapybio Biotechnology Co., Ltd.).

Trilineage endoderm, mesoderm, and ectoderm differentiation was performed using STEMdiff™ Trilineage specification Kit (STEMCELL Technologies, #05230). Briefly, hESCs were dissociated into single cells by TrypLE™ (ThermoFisher, 12604021) and resuspended in DMEM/F-12 (ThermoFisher, 11330057). After centrifuging at 300×*g* for 5 min, cell pellets were resuspended in mTeSR™1/Ectoderm Medium with 10 μM Y-27632. The Endoderm /Mesoderm/ Ectoderm Medium was replaced once a day. Endoderm and mesoderm lineages were harvested at day 5, while ectoderm lineage was harvested at day 7.

### Lentivirus package and infection

To package the sh1865 and sh2153 lentiviruses, the IFI16 target sequence (listed in Supplementary Table 1) was inserted into the hU6-MCS-CMV-Puromycin (GV112) plasmid. After transfecting HEK293T cells with sh1865/sh2153 GV112 with helper plasmids, the supernatant was collected at 48–72 h. Sequentially, the supernatant was filtered and centrifuged to harvest virus particles. The sh1865 and sh2153 lentiviruses were introduced to hESCs at MOI of 100. After infection, the sh1865 and sh2153 hESC lines were obtained by using 1 μg/ml puromycin.

To package the IFI16 overexpression lentivirus, the IFI16 sequence (NM_001206567, listed in Supplementary Table 1) was inserted into the TetIIP-MCS-3FLAG-Ubi-TetR-IRES-Puromycin (GV308) plasmid. After transfecting HEK293T cells with sh1865/sh2153 IFI16 overexpression GV308 with helper plasmids, the supernatant was collected at 48–72 h. Sequentially, the supernatant was filtered and centrifuged to harvest virus particles. The IFI16 overexpression lentivirus was introduced to hESCs at MOI of 300. After infection, the IFI16 overexpression hESC lines were obtained by using 1 μg/ml puromycin. And the IFI16 expression was induced by 1 μg/ml doxycycline.

### Immunofluorescence and image analysis

The prepared cells were washed twice with 0.1 mM phosphate-buffered saline (PBS) and then crosslinked by 4% paraformaldehyde for 20 mins at room temperature. After another wash with 0.1 mM PBS, the cells were incubated with 10% BSA and 0.5% Triton X-100 in PBS for 1 h. Primary antibodies (anti-IFI16 1:500, anti-AIM2 1:1000, anti-OCT4 1:1000, anti-SOX2 1:1000, anti-SOX17 1:1000, anti-FOXA2 1:500, anti-Brachyury 1:1000, anti-SNAI2 1:400, and anti-PAX6 1:1000) or isotypes (mouse IgG1/rabbit IgG 1:1000) were then added and incubated at 4 °C overnight. The next day, the cells were washed with 0.1 mM PBS three times and followed by incubation with secondary antibodies (1:1000) conjugated with a fluorophore at room temperature for 2–3 h The nucleus was then stained by using 4,6-Diamidino-2-phenylindole (DAPI). The fluorescence expression of OCT4, SOX2, SOX17, FOXA2, Brachyury, SNAI2, and PAX6 was defined as the average optical density (AOD) of immunoreactivity, which was quantified by ImageJ. Briefly, the integrated optical intensity of target gene was measured and then the background immunoreactivity was subtracted prior to analysis. For each sample, the background immunoreactivity was defined as the integrated optical intensity of the isotype (mouse IgG1/rabbit IgG) staining (Supplementary Figs. 11–13). The AOD in each sample was obtained by calculating the value of the average optical density normalized by integrated optical intensity of DAPI in the same field as following:

$${\mathrm{AOD}} = {\mathrm{IDT}}/{\mathrm{IDDT}} - {\mathrm{IDI}}/{\mathrm{IDDI}}$$

Where IDT is the integrated intensity of target gene, IDI is the integrated intensity of IgG and IDD is the integrated intensity of DAPI for target gene (IDDT) and isotype (IDDI).

### Flow cytometry

The marker protein of trilineage analyzed by flow cytometry was conducted as the manufacturer’s instructions of Fixation/Permeabilization Solution Kit (BD Biosciences, 554714). Briefly, the cultured cells were dissociated into single cells and resuspended in 0.1 mM PBS. After centrifuging at 300 × *g* for 5 min, cells were incubated with fixation/permeabilization solution for 20 min at room temperature. Sequentially, the diluted BD Perm/Wash™ Buffer was used to wash the cells twice. Then antibodies of marker protein (endoderm: SOX17-APC, FOXA2-488; mesoderm: Brachyury-APC, CXCR4-FITC; ectoderm: Nestin-APC, PAX6-488) or isotypes (mouse IgG2A-FITC, goat IgG-APC, goat IgG-488, mouse IgG-APC, mouse IgG2a kappa-488, Supplementary Fig. 14) were then added for staining. After staining at room temperature for 30 min, flow cytometry was then performed to analyze the 488/FITC^+^ and/or APC^+^ population. To evaluate the role of IFI16 in trilineage specification, the proportion of SOX17^+^/FOXA2^+^, Brachyury^+^/CXCR4^+^, Nestin^+^/PAX6^+^ cells was statistically analyzed after IFI16 knockdown and overexpression.

### RNA isolation, reverse transcription, and qPCR

Total RNA was extracted from the harvested cells by using Trizol (ThermoFisher, 15596026) and the concentration of the total RNA was measured by NanoDrop 2000 (ThermoFisher). The cDNA was then reverse-transcribed by using PrimeScript RT reagent Kit with gDNA Eraser (Takara, RR047A) from 1 μg RNA. The expression levels of genes were quantified by quantitative real-time PCR (qPCR) using AceQ Universal SYBR qPCR Master Mix (Vazyme, Q511-02). The reaction condition of qPCR: Denaturation at 95 °C for 2 mins. Denaturation at 94 °C for 15 s, annealing and extension at 60 °C for 30 s, 40 cycles. Quantification was analyzed by using the comparative Ct (∆∆Ct) method. The expression levels of genes were normalized by the housekeeping gene (GAPDH). The primer sequences for qPCR were listed in Supplementary Table 1.

### Western blots

The harvested cells were lysed in a lysis buffer (50 mM Tris-HCl (pH 6.8), 2% sodium dodecyl sulfate, 1.5% DL-Dithiothreitol, 10% glycerol and 0.2% Bromophenol blue), and denatured at 100 °C for 10 mins. Sequentially the total extracts were subjected to SDS-PAGE using 4–20% gradient gel (GenScript, M00655) and further transferred to Immobilon-FL PVDF (Millipore, IPFL85R). After blocking with Protein Free Rapid Block Buffer (EpiZyme, PS108), the protein was incubated with the primary antibodies (anti-IFI16 1:500, anti-AIM2 1:1000, anti-p53 1:500, anti-OCT4 1:1000, anti-SOX2 1:1000, anti-SOX17 1:1000, anti-FOXA2 1:1000, anti-Brachyury 1:1000, anti-OTX2 1:1000, anti-PAX6 1:1000, and anti-β-actin 1:5000) at 4 °C overnight. The next day, the protein was washed with Tris-buffered saline +0.1% Tween-20 (TBST) three times and then incubated with secondary antibodies for 2 h at room temperature. The expression of protein was detected by chemiluminescence imaging system (GeneGnomeXRQ, SYNGENE) and analyzed by densitometry using Image J. The background correction was done with the value of 50 (called rolling disc in the software). To calculate the relative expression of specific protein, the β-actin serves as a reference for the sample loading. All blots derive from the same experiment and were processed in parallel. The uncropped blots are listed in the Supplementary Figs. 15–23.

### Co-Immunoprecipitation

The hESCs and differentiated trilineage cells were cultured in a 10 cm dish for 2 days. Sequentially, the cells were harvested and lysed in Pierce™ IP Lysis Buffer (ThermoFisher, 87787). After rotation at 4 °C for 1 h, the cell lysates were incubated with p53 or IFI16 antibodies at 4 °C overnight. The next day, the protein A/G magnetic beads (Biotool, b23202) were added to capture the protein-antibody complex at 4 °C for 3 h After washing twice with Pierce™ IP Lysis Buffer and a final wash with TBST, the magnetic beads were incubated in western blots lysis buffer at 100 °C for 10 mins to elute the target protein. Lastly, the eluted products were subjected to western blots analysis.

### PLA technology

The in situ p53-IFI16 interaction was detected using Duolink® In Situ Red Starter Kit Mouse/Rabbit (Sigma, DUO92101) according to the manufacturer’s instructions. Briefly, the cultured cells were washed with 0.1 mM PBS and then crosslinked by 4% paraformaldehyde for 15 min at room temperature. After blocking the cells with the Duolink® Blocking Solution for 1 h at 37 °C, primary antibodies (anti-IFI16 1:500 and anti-p53 1:500) were then added and incubated at 4 °C overnight. The next day, the cells were washed with 2 × 5 mins in 1× Wash Buffer A at room temperature and followed by incubation with the proximity ligation assay (PLA) probe solution at 37 °C for 1 h. After another 2 × 5 min-wash with 1× Wash Buffer A, the ligation solution was applied at 37 °C for 30 min. Sequentially, the ligation solution was removed and the cells were then washed with 2 × 5 mins in 1× Wash Buffer A. After incubation with the amplification at 37 °C for 2 h, the final wash with 1× Wash Buffer B was performed. The nucleus was then stained by using Duolink® In Situ Mounting Medium with DAPI. The fluorescence signals were detected using a confocal microscope (Leica, TCS SP8 X).

### Alkaline phosphatase staining

The alkaline phosphatase staining was conducted as the protocol of Alkaline Phosphatase Kit (Sigma, 85L2). Briefly, the cultured cells were fixed using the Fixative Solution for 30 s at room temperature. After rinsing gently in deionized water for 45 s, the alkaline-dye mixture was applied at room temperature for another 30 min. Sequentially, the cells were rinsed gently in deionized water for 2 min and then incubated with Mayer’s Hematoxylin Solution for 10 mins at room temperature.

### CCK-8 assay

The effect of IFI16 knockdown on cell viability was determined with Cell Counting Kit-8 (CCK-8) assay (MedChemExpress, HY-K0301). Briefly, the sh1865 and shNC hESC lines were dissociated into single cells and further induced into trilineage using STEMdiff™ Trilineage specification Kit in 24-well plates. The CCK-8 solution was added to each well of the plate at Day 0, Day 1, Day 2, and Day 5 for the endoderm and mesoderm, Day 0, Day 1, Day 2, and Day 7 for the ectoderm. After incubation for 2 h, the supernatant was collected and the absorbance was measured at 450 nm.

### ChIP-seq

The cultured cells (about 5 million) were crosslinked with 1% formaldehyde for 5 min at room temperature, and then quenched with 125 mM glycine. After a wash with 0.1 mM PBS, the cells were harvested and lysed in ChIP Lysis Buffer (0.1% SDS, 1% Triton X-100, 2 mM EDTA, 150 mM NaCl, 50 mM Tris-HCl pH 8.0) at 4 °C for 1 h to lyse the cell membrane. After centrifuging at 2500×*g* for 5 mins, the nuclei were collected and the chromatin DNA was sheared into 300–600 bp fragments by the sonicator (protocol: 25% amplitude; duration of 15 min; 30 s ON and 30 s OFF; JY99-IIDN, SCIENTZ). Sequentially, the supernatant was harvested after centrifuging at 12,000×*g* for 10 mins. The antibodies (p53 or c-Jun, 10 μg) were added for incubation at 4 °C overnight. Next day, the protein A/G magnetic beads were added to capture the protein-antibody complex at 4 °C for 3 h. After careful washes with Low Salt Wash Buffer (0.1% SDS, 1% Triton X-100, 2 mM EDTA, 50 mM HEPES pH 7.9, 150 mM NaCl), High Salt Wash Buffer (0.1% SDS, 1% Triton X-100, 2 mM EDTA, 50 mM HEPES pH 7.9, 500 mM NaCl), LiCl Wash Buffer (250 mM LiCl, 0.5% NP40, 0.5% sodium deoxycholate, 1 mM EDTA, 20 mM Tris-HCl pH 8.0,), and TE Buffer (10 mM Tris-HCl pH 8.0, 1 mM EDTA), the was ChIP products were eluted with ChIP Elution Buffer (1% SDS, 10 mM EDTA, 50 mM Tris-HCl pH 8.0) at 65 °C for 20 min. Crosslinking was reversed in 65 °C overnight and the protein was digested by proteinase K (20 mg/ml). ChIP-seq library construction and further sequencing was carried out by Annoroad Gene Technology. For ChIP-qPCR, the DNA binding levels of c-Jun at IFI16 promoter, AIM2 promoter, cGAS promoter and IFI16 3’UTR during trilineage specification were calculated relatively to those of H9 cells. The c-Jun binding sites of AIM2 promoter and cGAS promoter is referred to the UCSC Genome Browser (http://genome.ucsc.edu/).

### RNA-seq

For gene expression level measurement, total RNA was extracted from the hESCs and trilineage differentiated cells using TRIzol. RNA-seq library construction and further sequencing was carried out by Annoroad Gene Technology.

### ChIP-seq and RNA-seq data analysis

After the quality and redundancy filtering, the sequenced tags were used for peak calling using the MACS2 algorithm with the settings for peak enrichment >20, peak-to-background enrichment >3, a kernel bandwidth of 300. The p53 ChIP-seq peaks were analyzed using the MEME using a de novo motif analysis of the binding site sequences with default parameters. TOMTOM tool of MEME was used for alignment of the achieved DNA motifs with the identified motifs in the JASPAR database. The comparisons between the shNC and sh1865 p53 ChIP-seq peaks were performed using MAnorm with default parameters.

For the RNA-seq analysis, the alignments were performed using the STAR aligner, and the differential expression analysis was conducted using DESEQ2 as suggested to obtain a list of differentially regulated genes (Fold change >1.5, FDR < 0.01).

The biological functions of the genes derived from these p53 ChIP-seq peaks and differentially regulated genes during trilineage differentiation were annotated by using ‘BiNGO’ (Biological Network Gene Ontology) tool of Cytoscape for biological processes gene ontology (GO) terms, with significance threshold 0.01. Each node represented a specific GO term, and the node size indicated the number of genes in the GO term.

The enhancer identification was conducted using human enhancer database from FANTOM5 (http://fantom.gsc.riken.jp/papers/). Briefly, the p53 peaks of TSS upstream (2 to 100 kb) of p53-activated and p53-repressed genes in differentiated trilineage were harvested, respectively, to align with the validated enhancer sequences in the FANTOM5. The enhancer that overlaps with p53 peak was retained, and the enhancer that falls in the intron, exon and UTRs of genes was further rejected.

### Antibodies and reagents

Antibodies were used as following: IFI16 was purchased from Abcam (ab169788, Western blots/Co-IP) and Santa Cruz (sc-8023, PLA/Immunofluorescence), p53 was purchased from CST (2527, PLA) and Santa Cruz (sc-126, ChIP/Western blots/Co-IP), AIM2 was purchased from Abcam (ab180665, Western blots/Immunofluorescence), cGAS was purchased from CST (15102S, Western blots/ Immunofluorescence), OCT4 was purchased from Abcam (ab19857, Western blots/Immunofluorescence), SOX2 was purchased from CST (2748S, Western blots/Immunofluorescence), Brachyury was purchased from Abcam (ab20680, Western blots/ Immunofluorescence), OTX2 was purchased from Abcam (ab21990, Western blots/ Immunofluorescence), PAX6 was purchased from Abcam (ab5790, Western blots/Immunofluorescence), SOX17 was purchased from Abcam (ab84990, Western blots/ Immunofluorescence), SNAI2 was purchased from Santa Cruz (sc-166476, Immunofluorescence), FOXA2 was purchased from R&D (AF2400, Western blots/Immunofluorescence), c-Jun was purchased from Abcam (ab31419, Western blots), p38 was purchased from CST (9212s, Western blots), p-p38 was purchased from CST (9211s, Western blots), ERK was purchased from CST (4695s, Western blots), p-ERK was purchased from CST (4370s, Western blots), JNK was purchased from CST (9252s, Western blots), p-JNK was purchased from Santa Cruz (sc-6254, Western blots), CXCR4-FITC was purchased from R&D (FAB170F, Flow cytometry), Brachyury-APC was purchased from R&D (IC2085A, Flow cytometry), SOX17-APC was purchased from R&D (IC1924A, Flow cytometry), FOXA2-488 was purchased from R&D (IC2400G, Flow cytometry), Nestin-APC was purchased from R&D (IC1259A, Flow cytometry), PAX6-488 was purchased from BD (561664, Flow cytometry), β-actin was purchased from ZEN BIO (200058-BF10, Western blots), rabbit IgG isotype control was purchased from CST (3900S, Immunofluorescence), mouse IgG1 isotype control was purchased from CST (5415S, Immunofluorescence), mouse IgG2A-FITC was purchased from R&D (IC003F, Flow cytometry), goat IgG-APC was purchased from R&D (IC108A, Flow cytometry), goat IgG-488 was purchased from R&D (IC108G, Flow cytometry), mouse IgG1-APC was purchased from R&D (IC002A, Flow cytometry), and mouse IgG2a kappa-488 was purchased from BD (565358, Flow cytometry).

Drugs were used as following: SP600125, SB203580, and U0126 were all purchased from Selleck Chemicals (S1460, S1076, and S1102).

### Statistical analysis

Statistical analysis was carried out using GraphPad Prism 5.0 evaluate the differences between different groups. All data were presented as mean ± SEM from three or more independent experiments. Comparisons between groups for statistical significance were performed with Student’s *t* test or analysis of variance (ANOVA) with post hoc test in multiple groups. Results were considered significant difference at *P < 0.05; **P < 0.01; ***P < 0.001; ^#^*P* < 0.05; ^##^*P* < 0.01; ^###^*P* < 0.001, respectively.

### Reporting summary

Further information on research design is available in the Nature Research Reporting Summary linked to this article.

## Supplementary information

Supplementary Information

Supplementary Dataset 1

Reporting Summary

## Data Availability

All relevant data of this study are available within the paper. ChIP-seq and RNA-seq raw data and processing data supporting this work have been deposited in Gene Expression Omnibus under the accession number GSE142050. Requests for further information should be directed to and will be fulfilled by Furong Li (frli62@163.com).

## Code availability

All studies used the commercial software in the generation or processing of datasets. Software and the corresponding version numbers were listed as following: ImageJ 1.46j, Photoshop CS6, Illustrator Adobe CS6, Graphpad Prism 5.0, FlowJo 10.4.2, MACS2 2.2.1, MEME 5.1.0, MAnorm 1.2.0, Cytoscape 3.7.1, DESEQ2 3.10, FANTOM5 5.
