# Supplementary material for: IFI16 promotes human embryonic stem cell trilineage specification through interaction with p53
Source: NPJ Regen Med. 2020 Oct 29;5:18. doi: 10.1038/s41536-020-00104-0 (PMC7596047; doi:10.1038/s41536-020-00104-0)
Supplement: Supplementary file 1 — Supplementary Information [file 41536_2020_104_MOESM1_ESM.pdf]

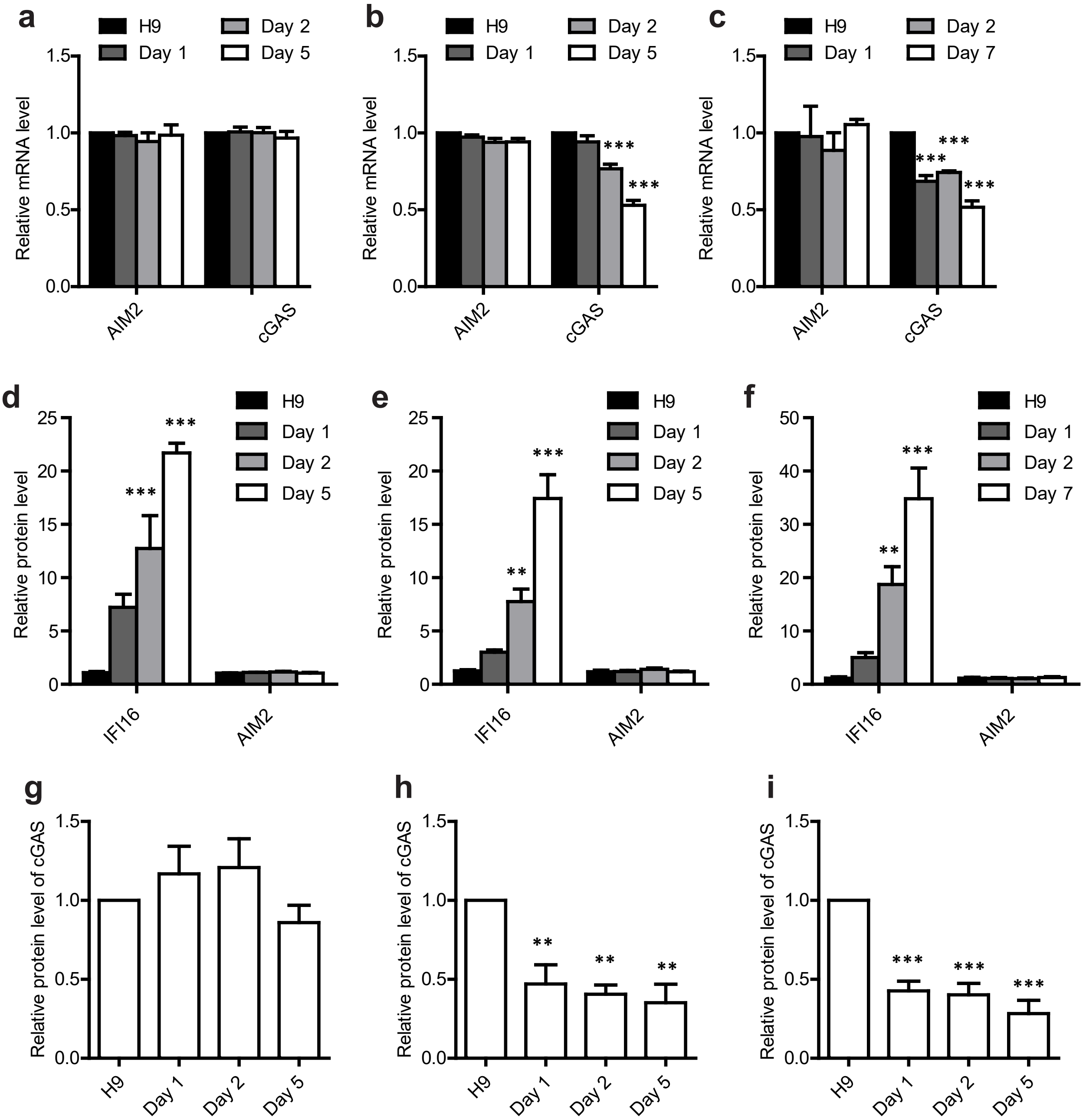

Supplementary Figure. 1

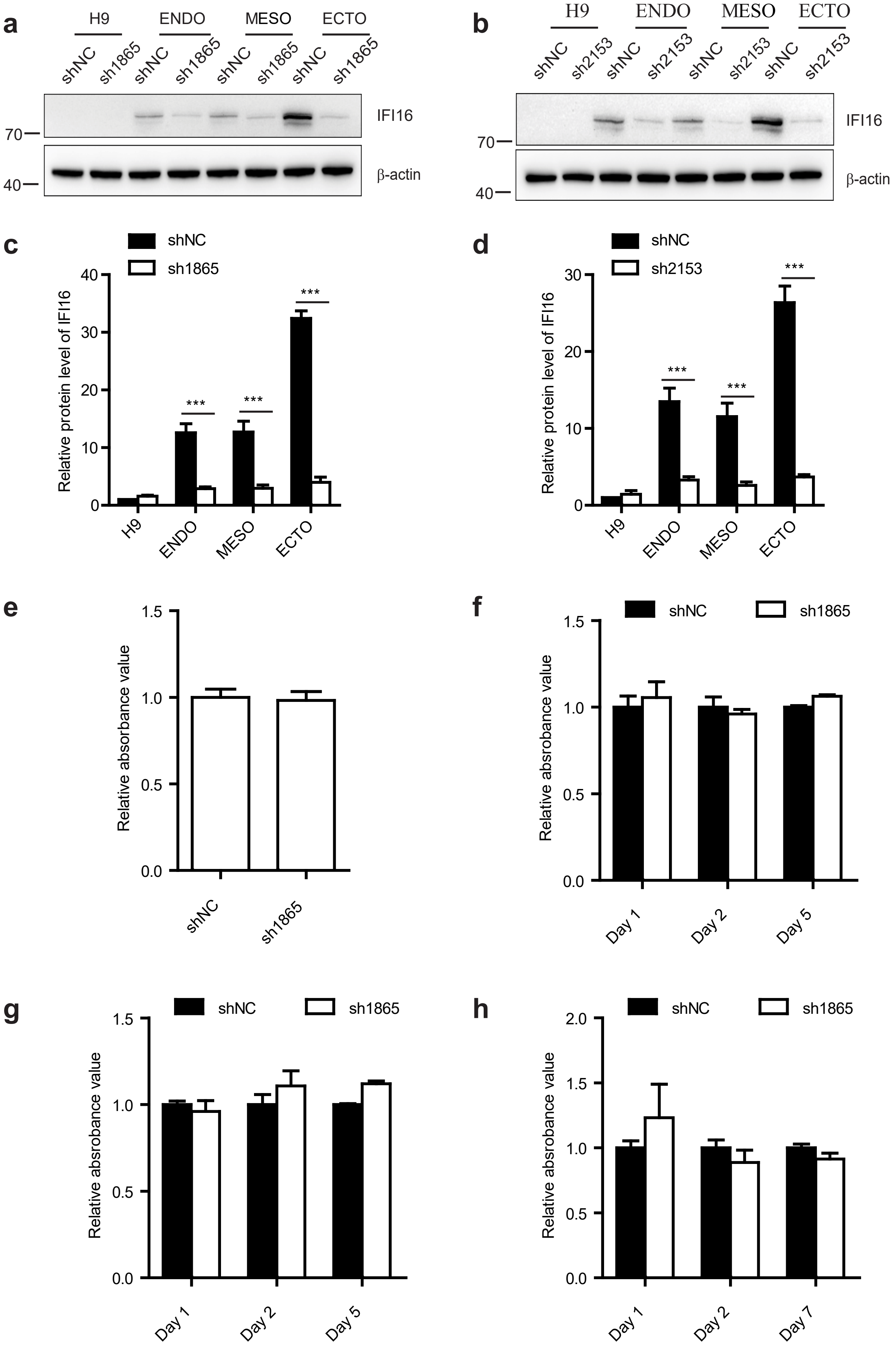

Supplementary Figure. 2

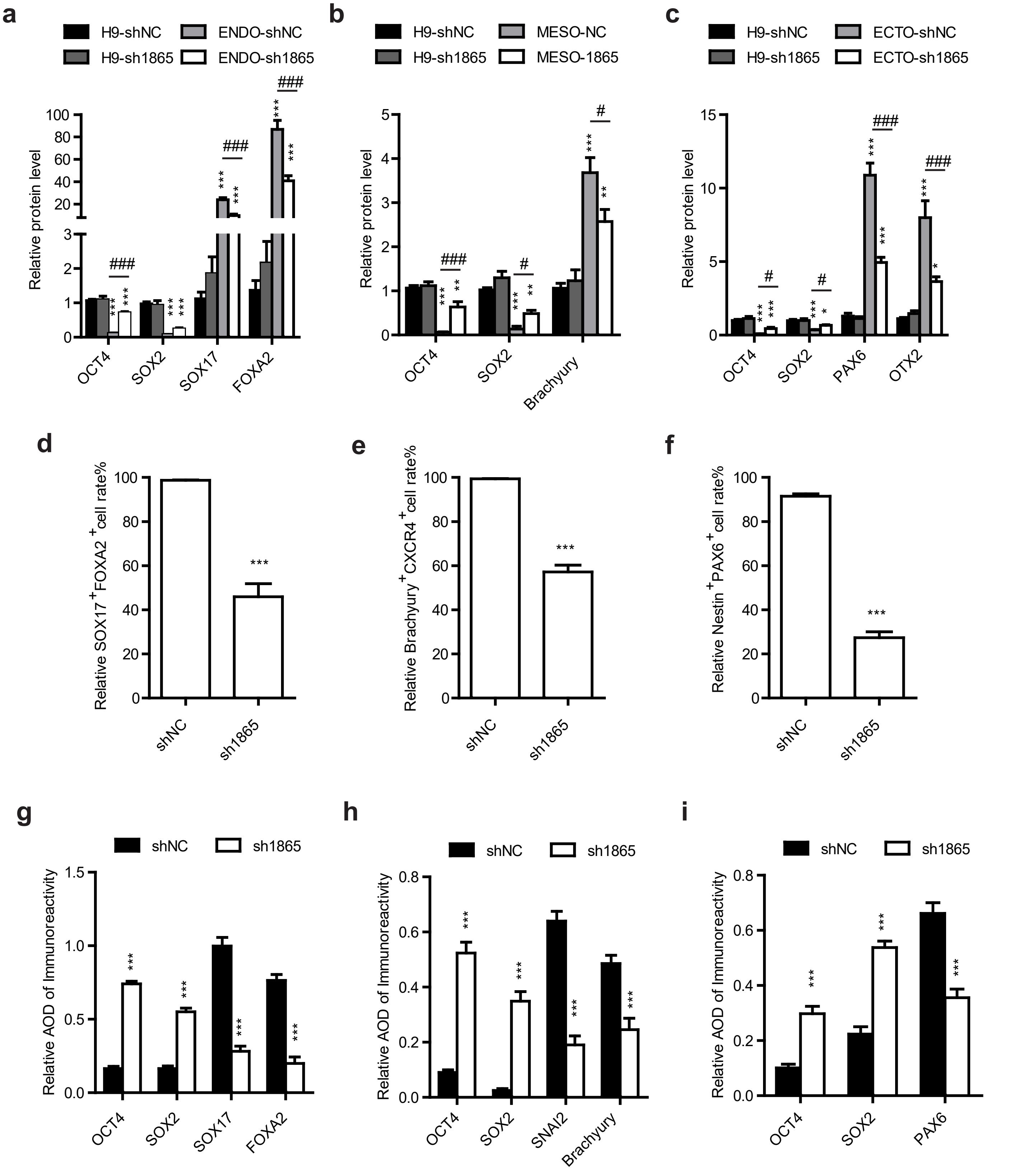

Supplementary Figure. 3

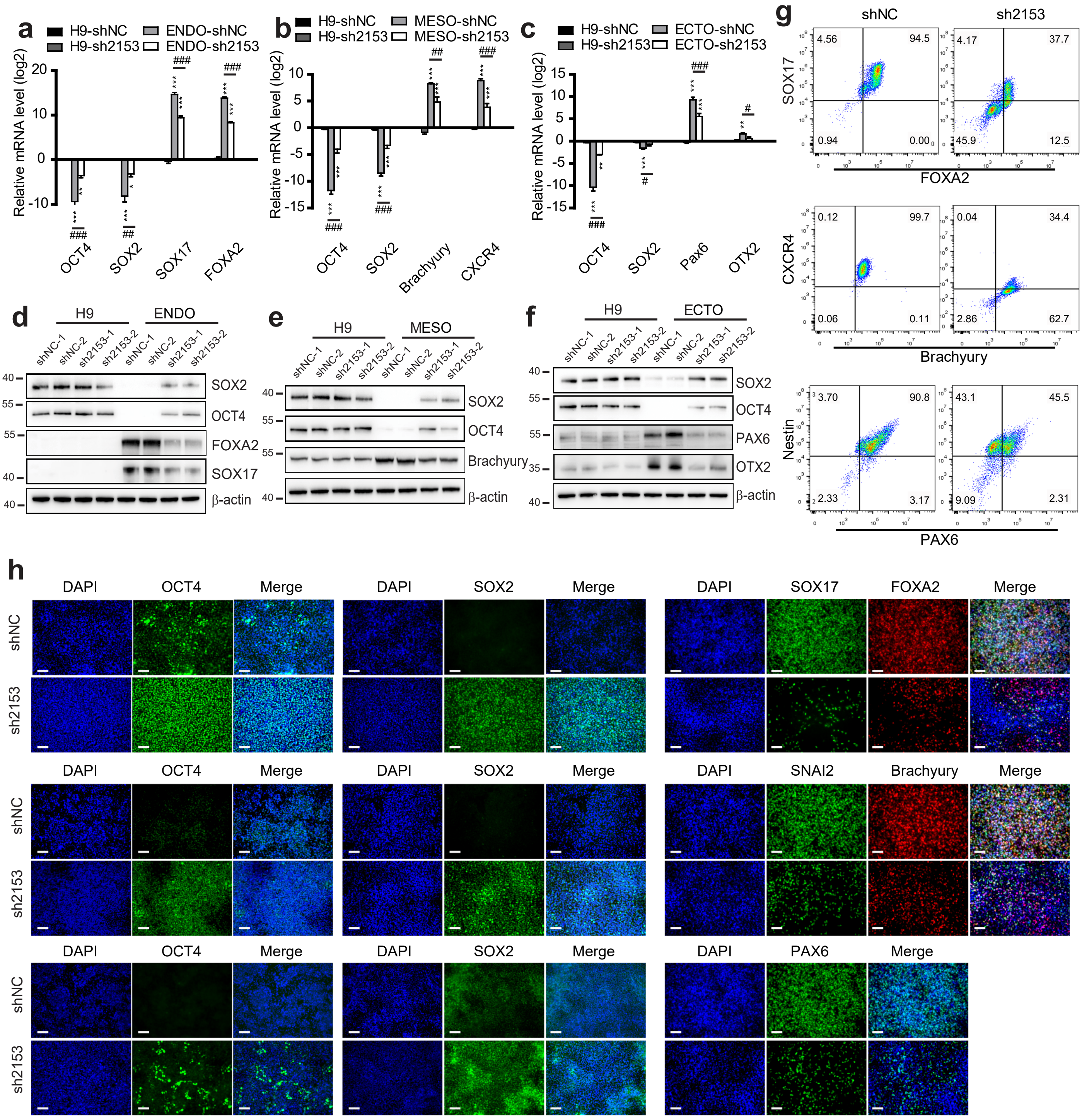

Supplementary Figure. 4

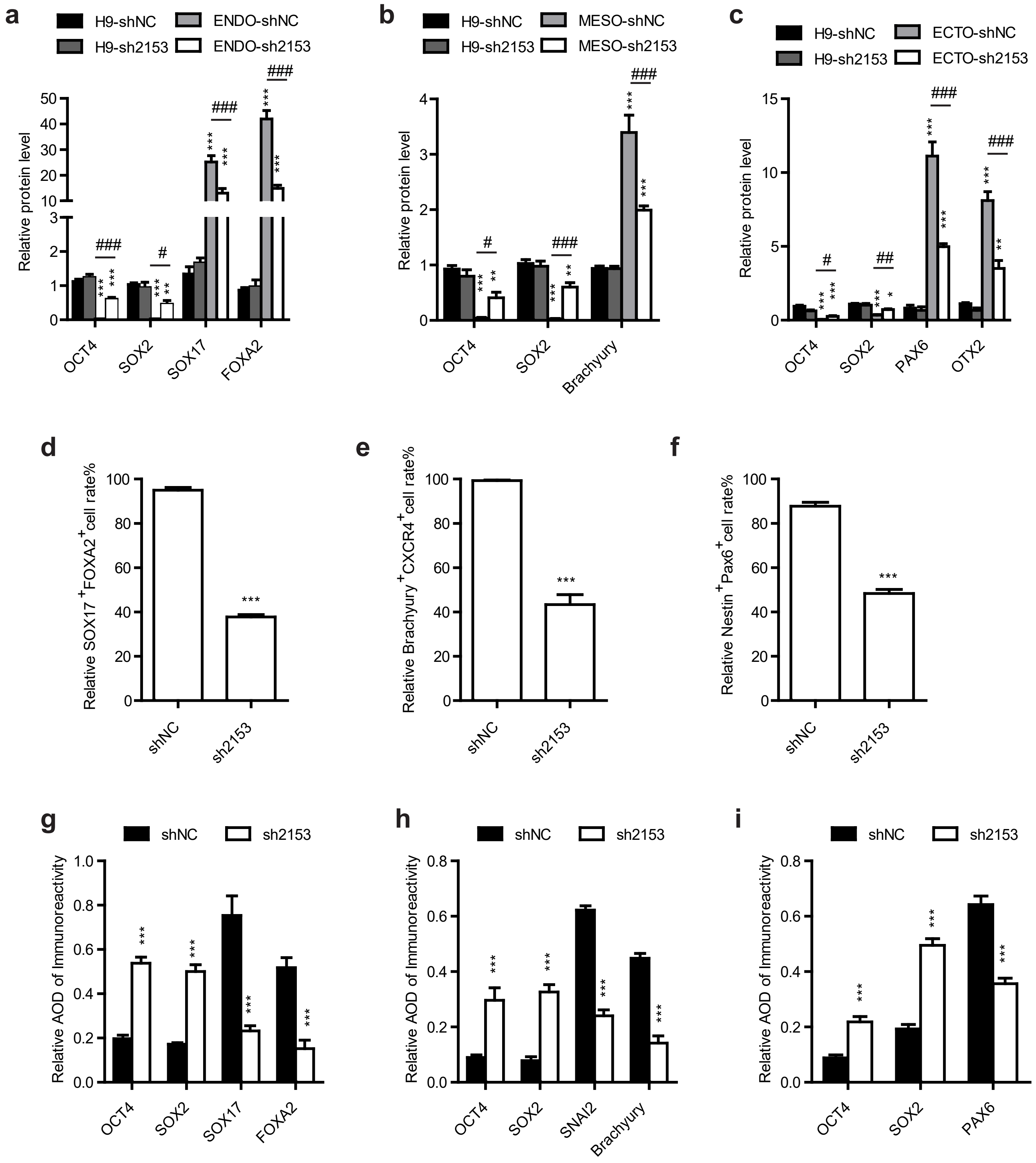

Supplementary Figure. 5

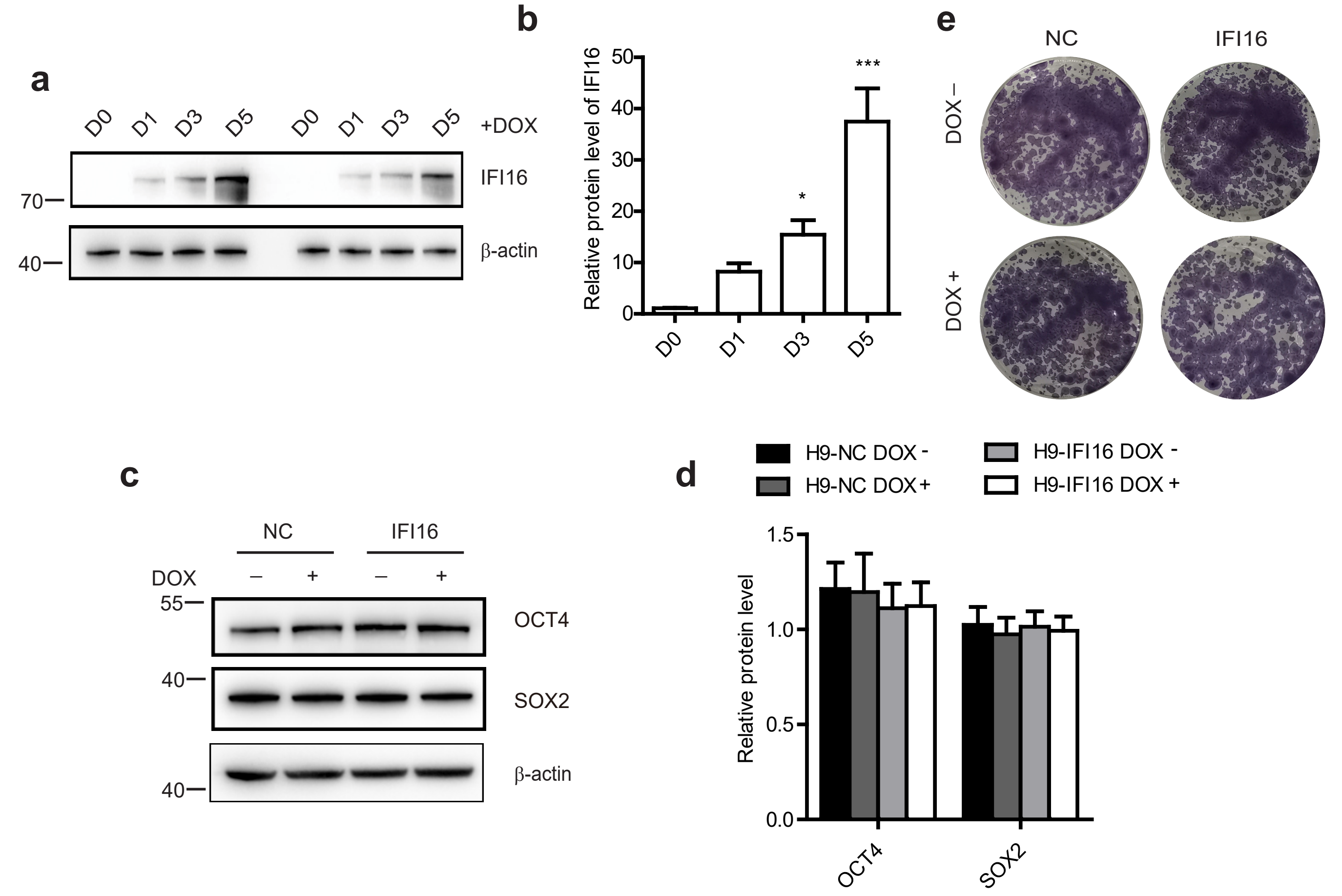

Supplementary Figure. 6

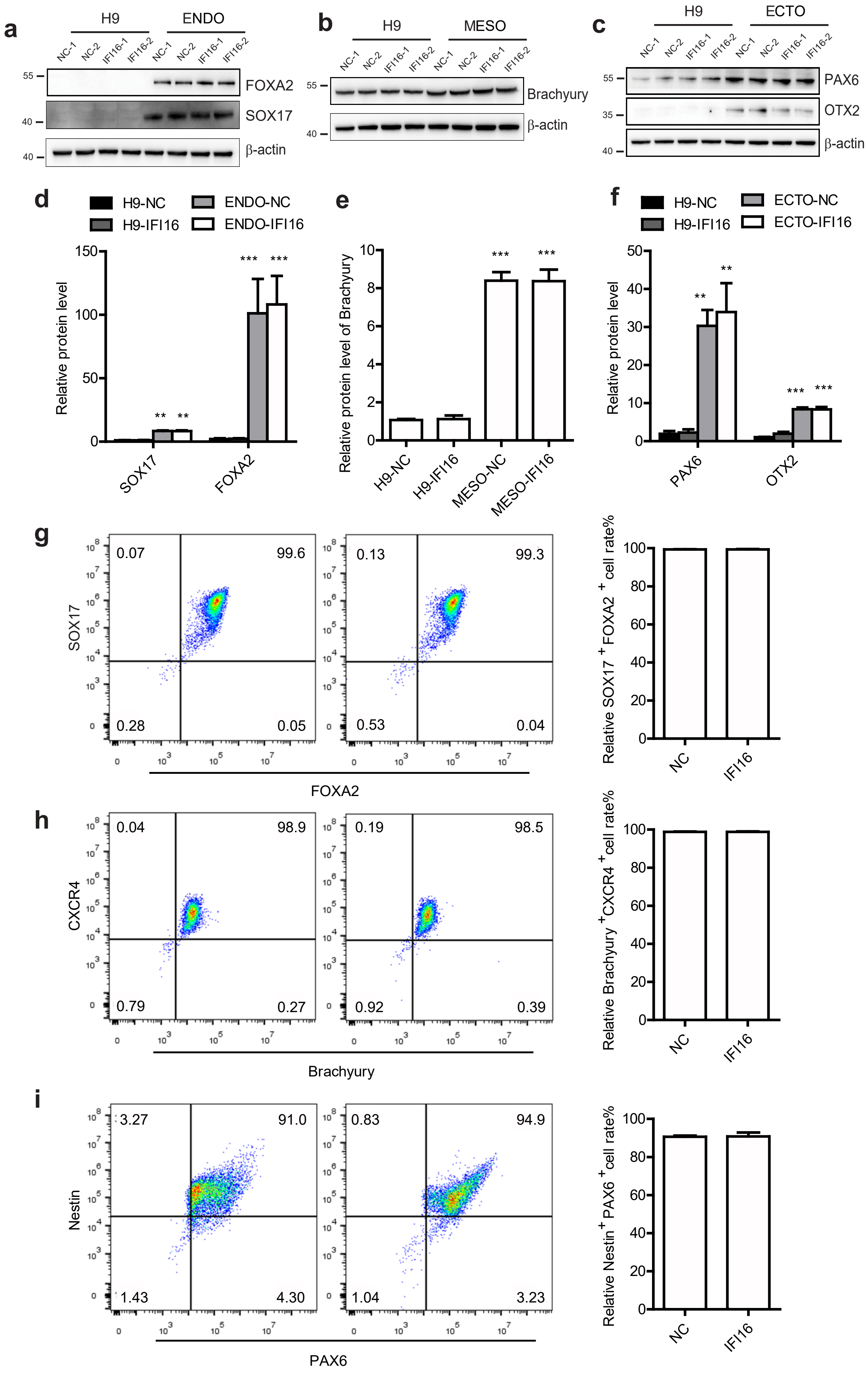

Supplementary Figure. 7

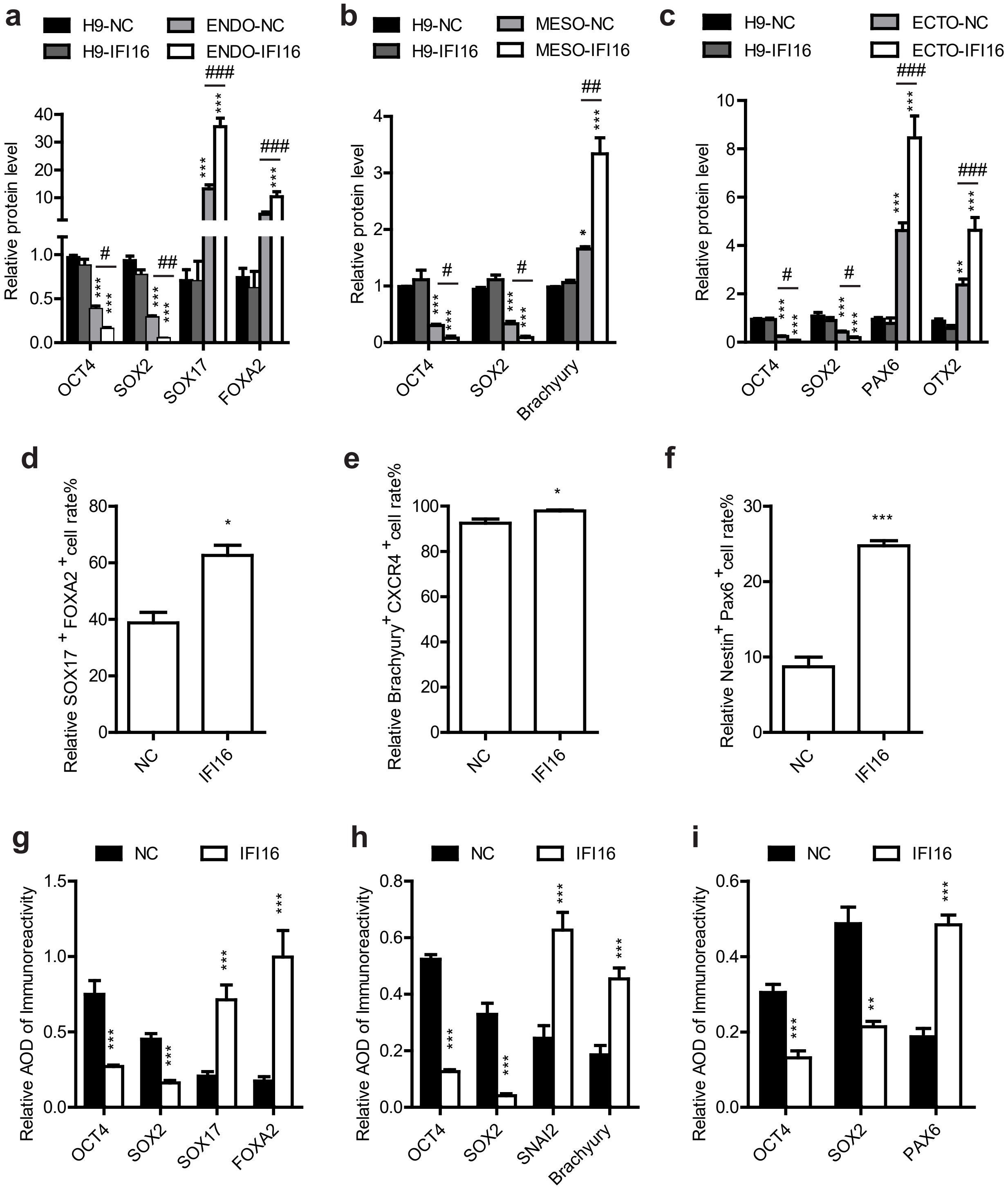

Supplementary Figure. 8

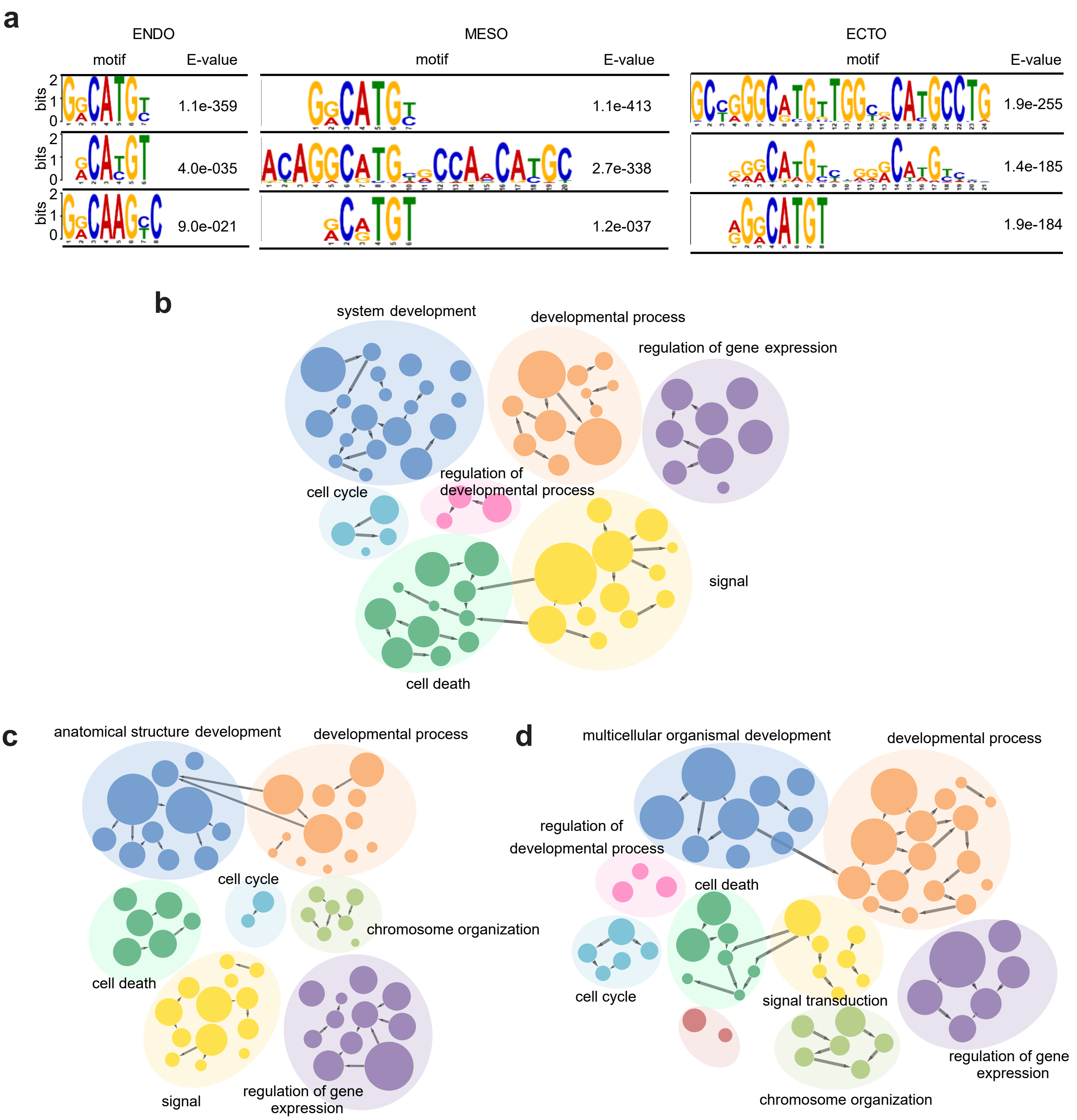

Supplementary Figure. 9

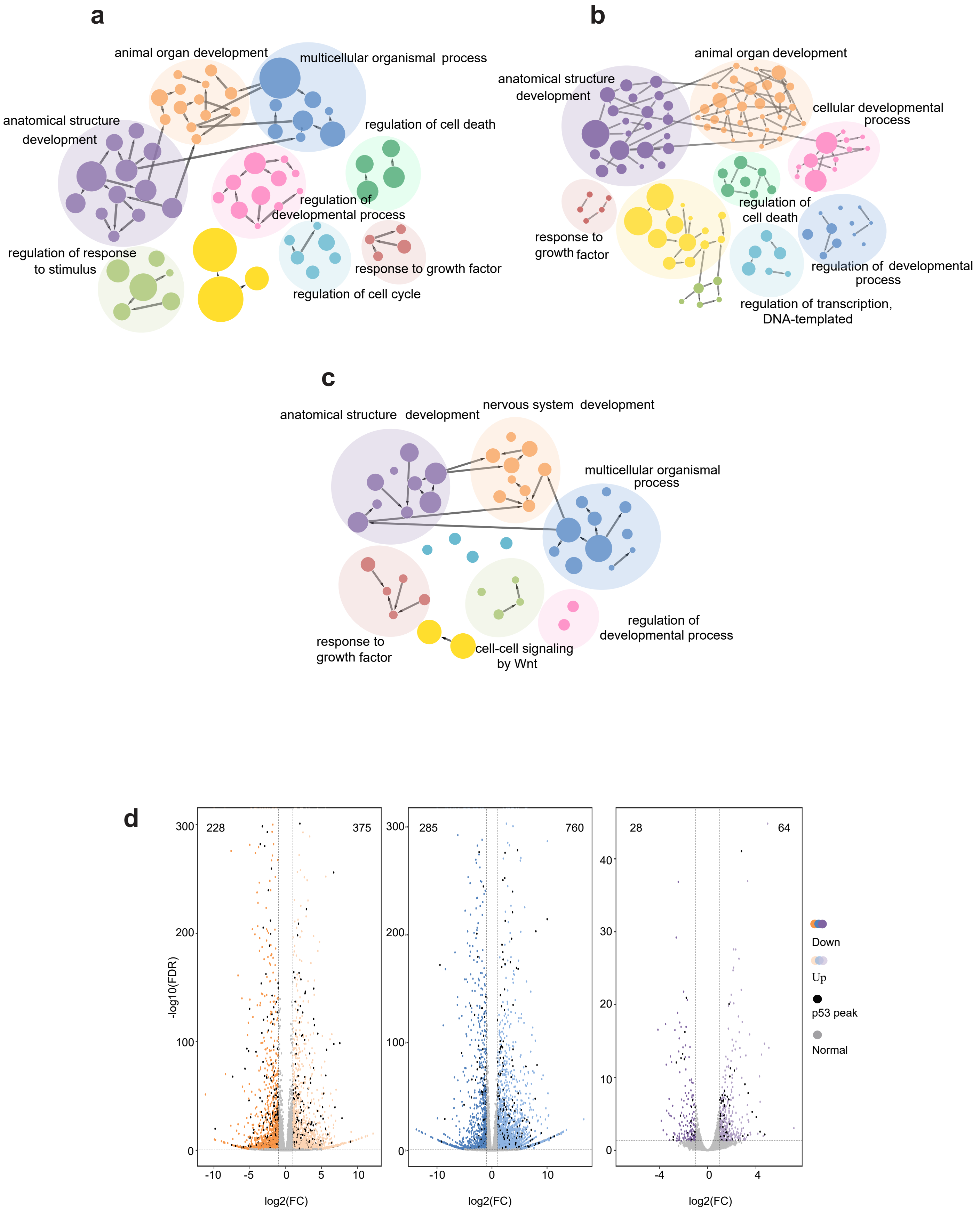

Supplementary Figure. 10

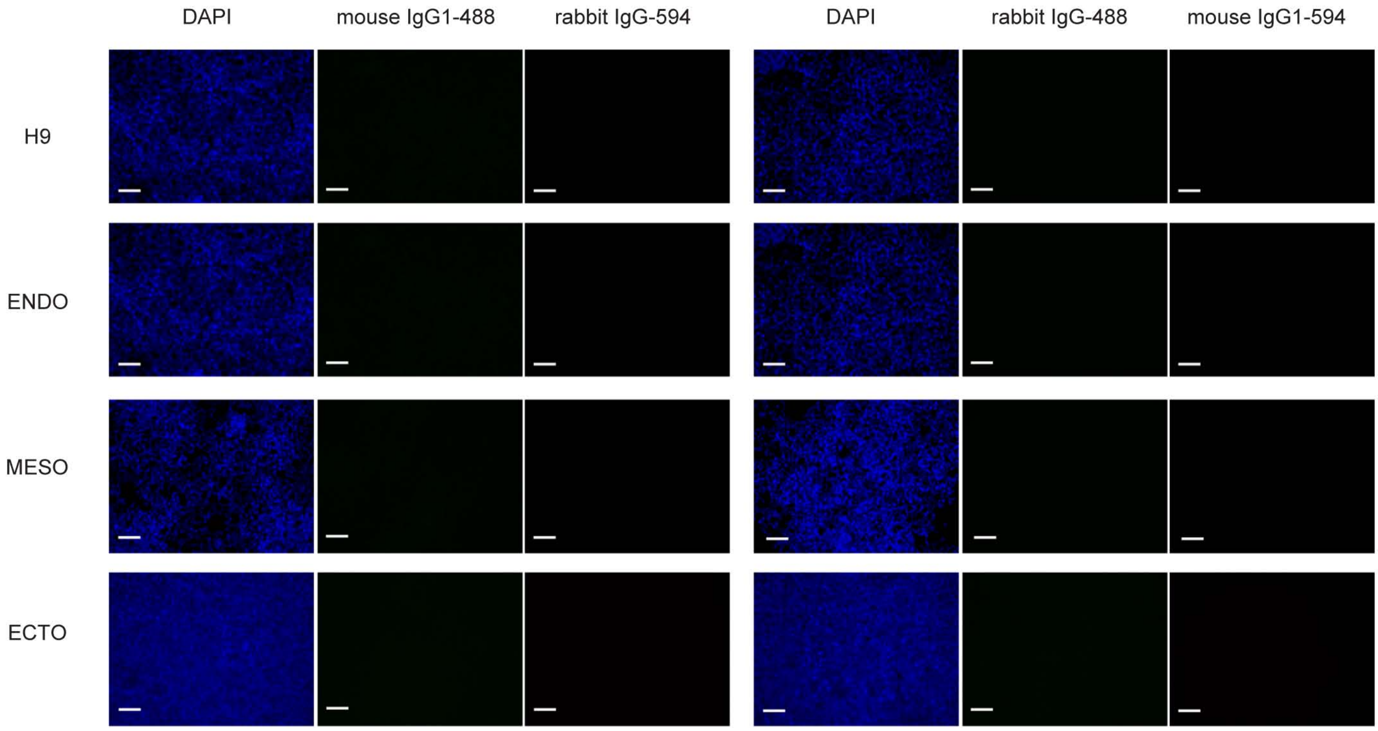

Supplementary Figure. 11

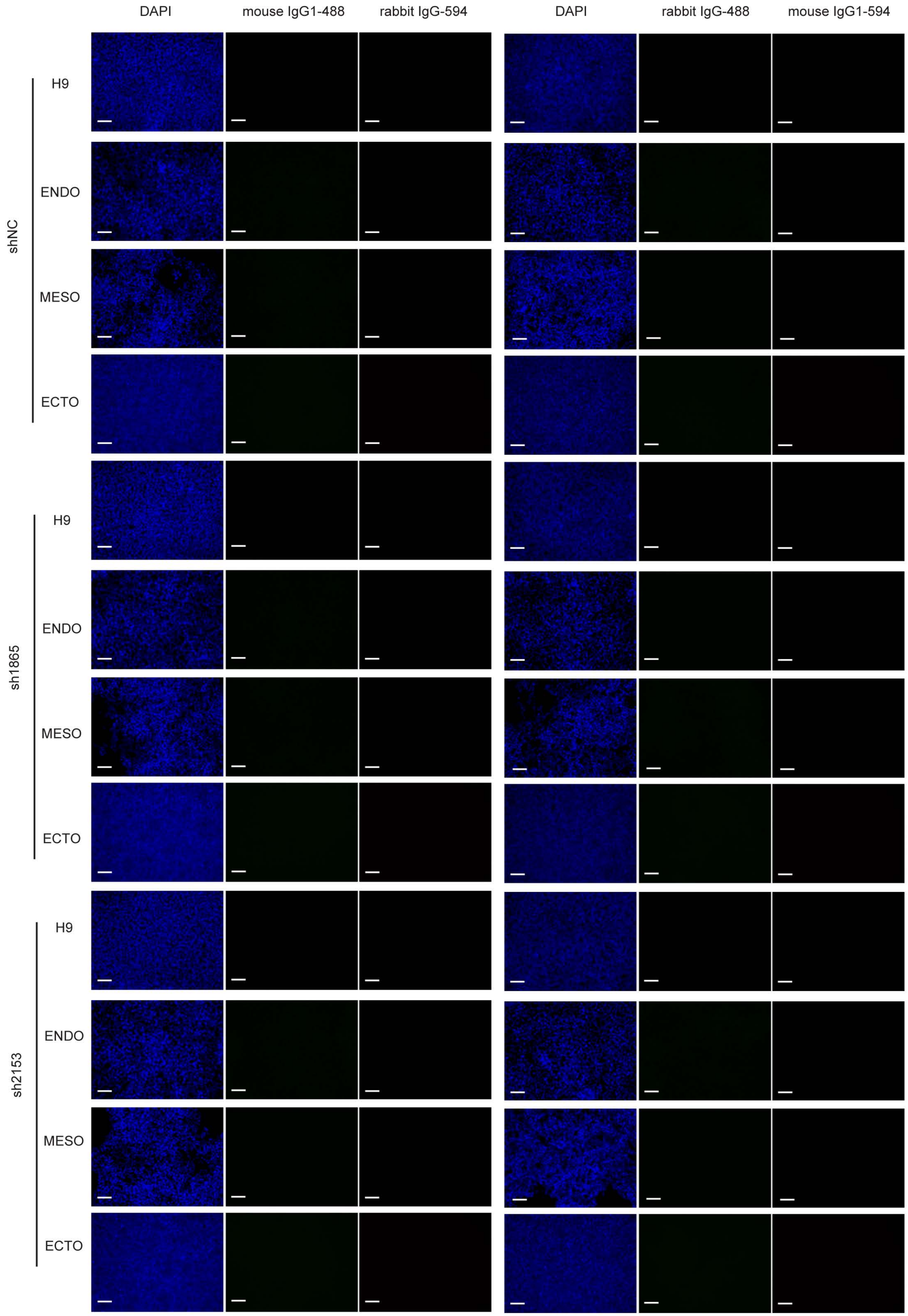

Supplementary Figure. 12

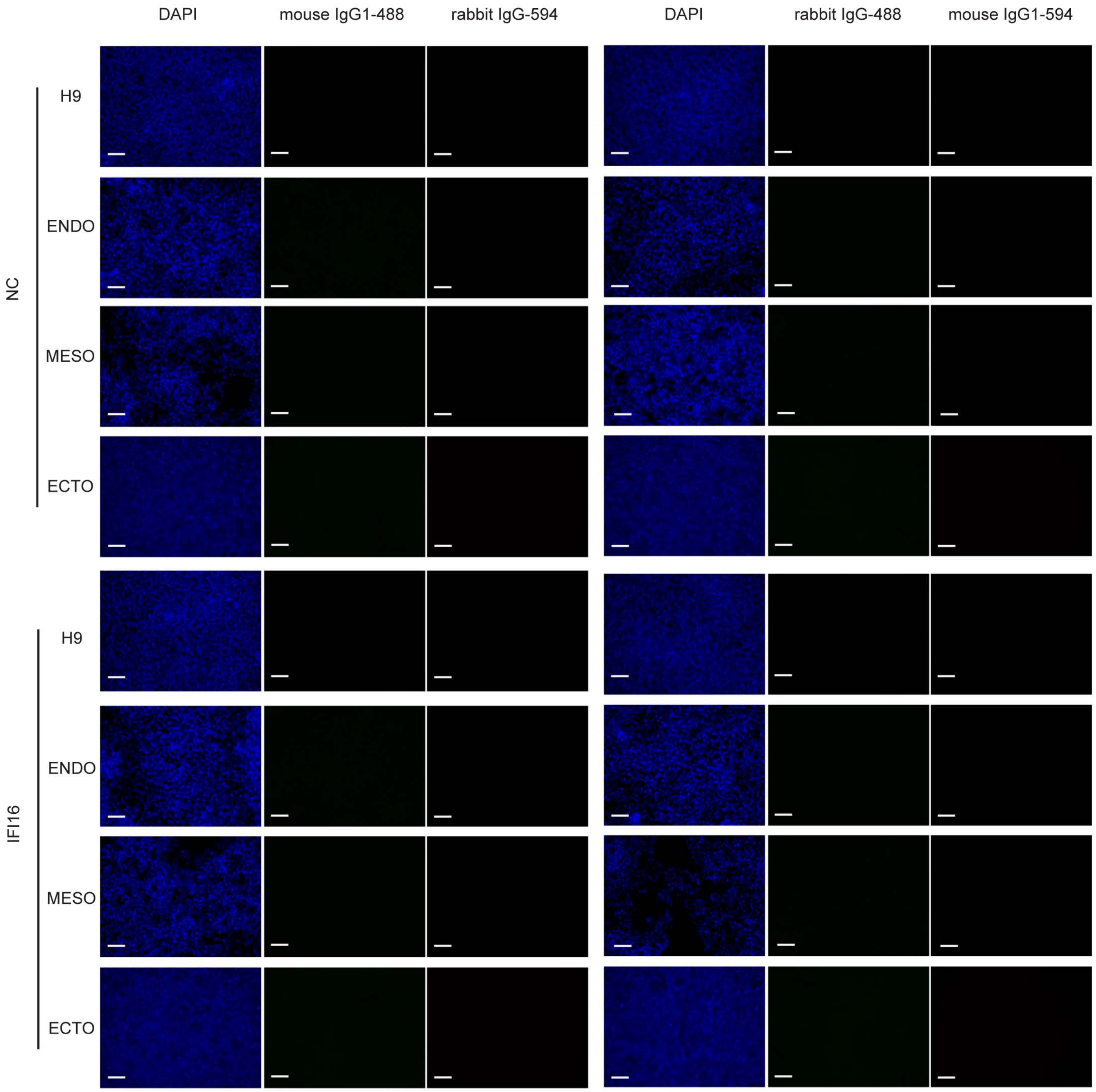

Supplementary Figure. 13

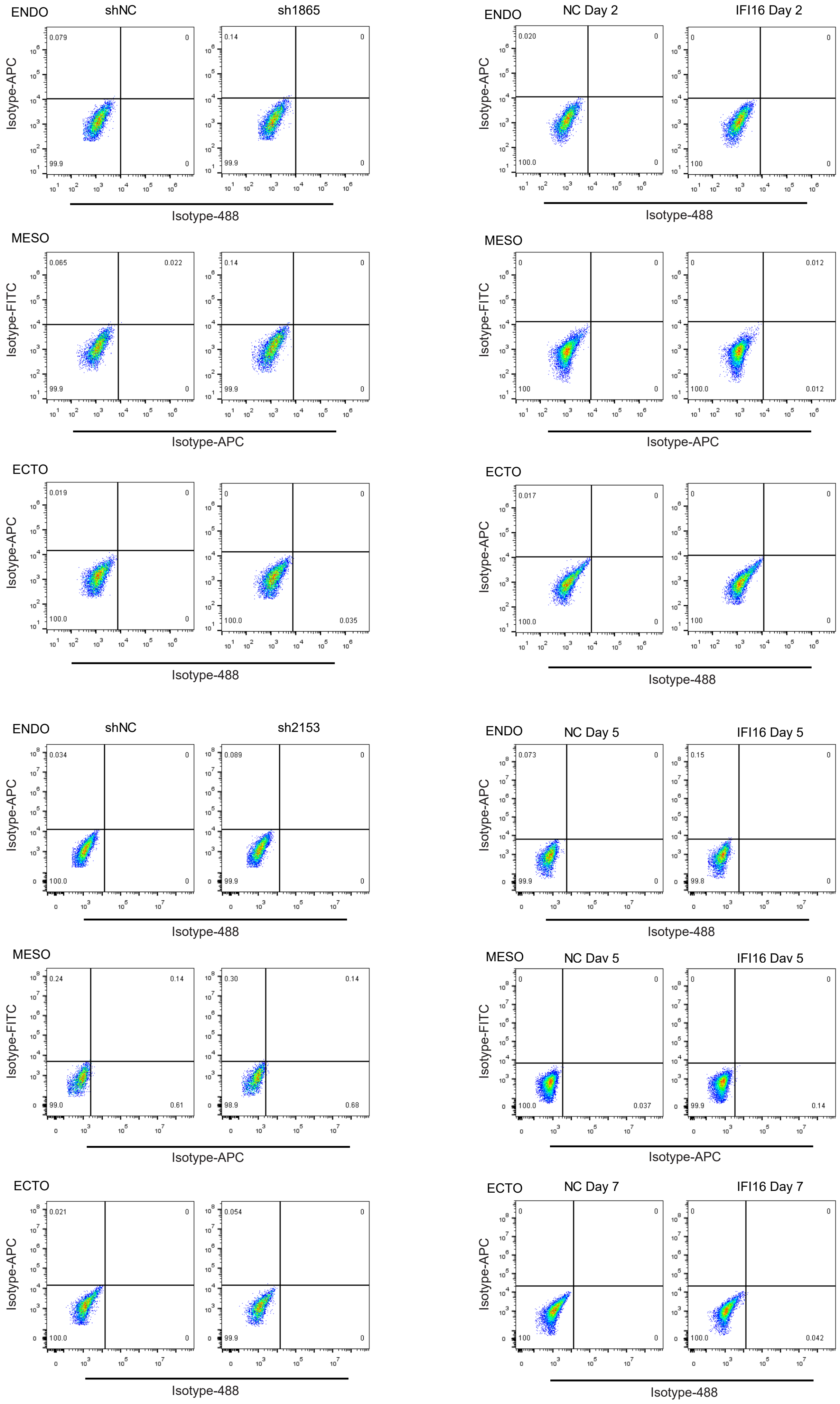

Supplementary Figure. 14

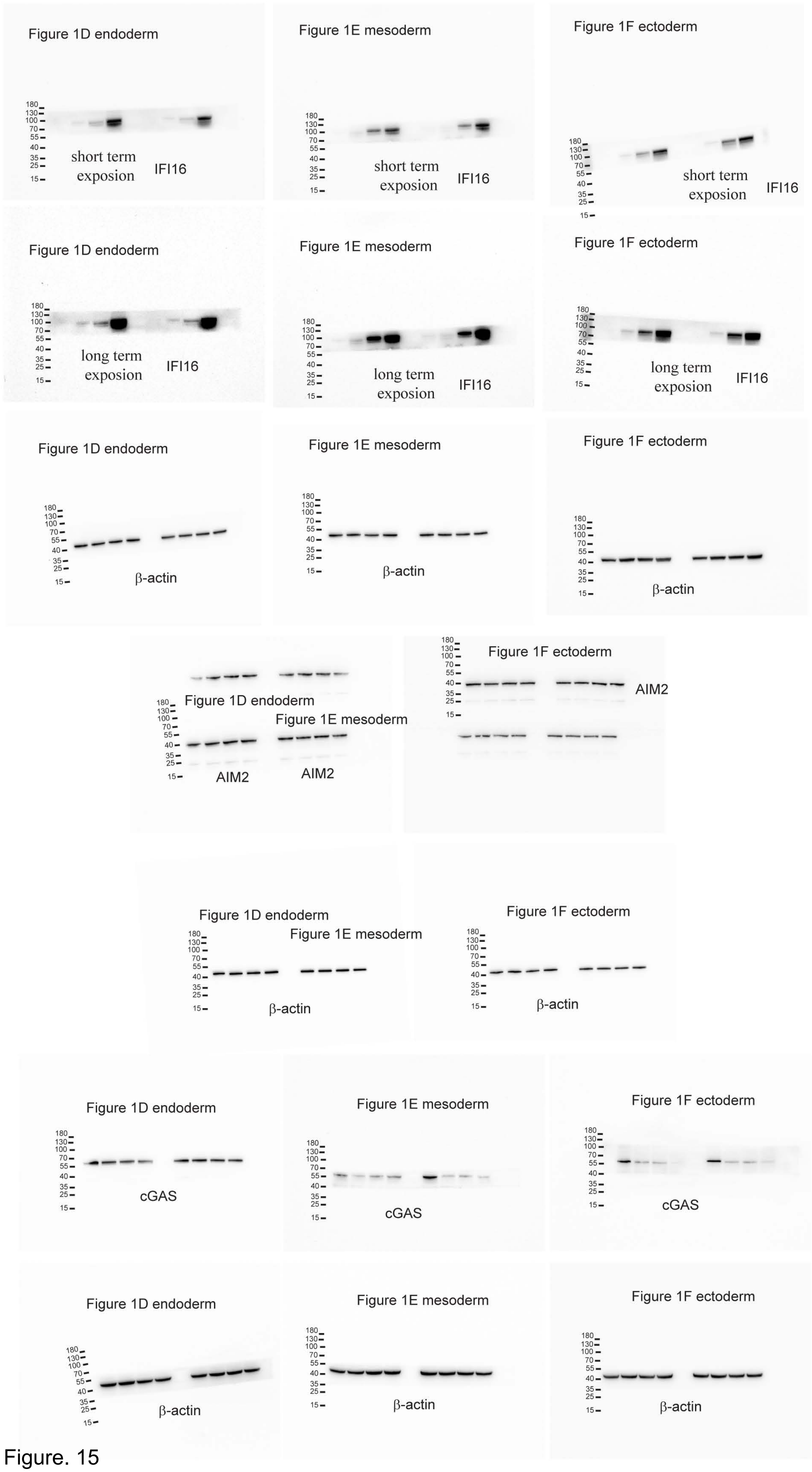

Supplementary Figure. 15

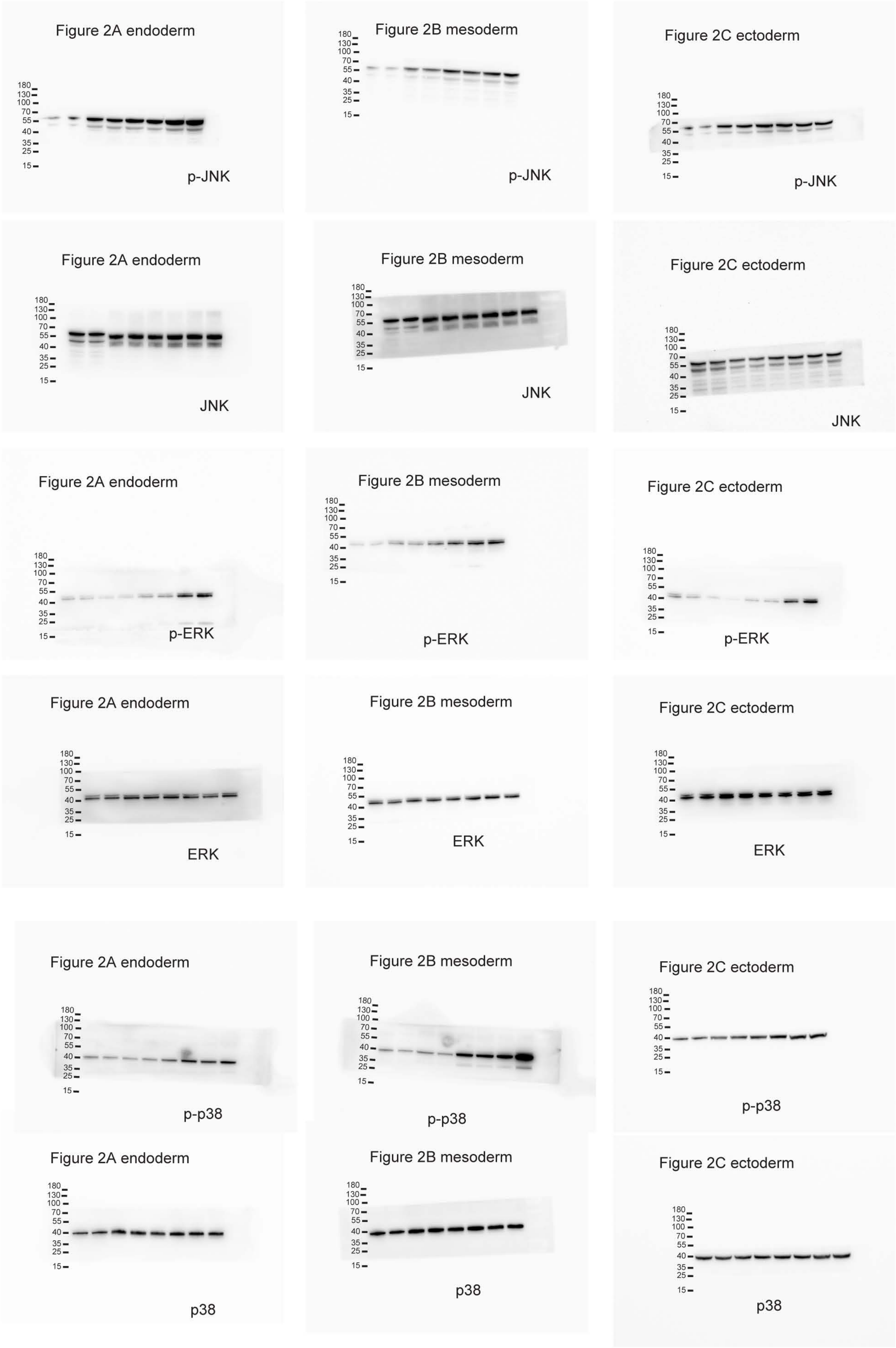

Supplementary Figure. 16

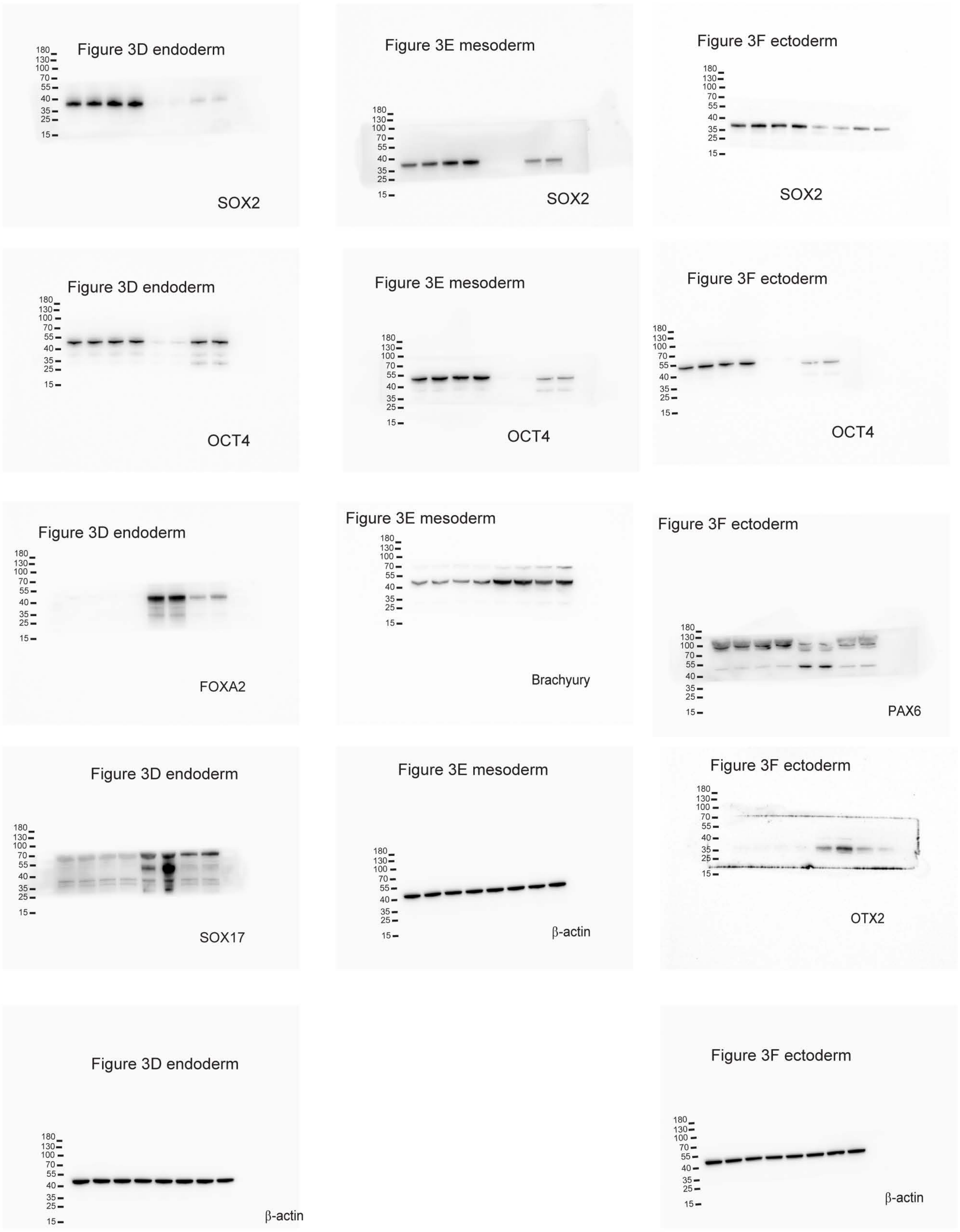

Supplementary Figure. 17

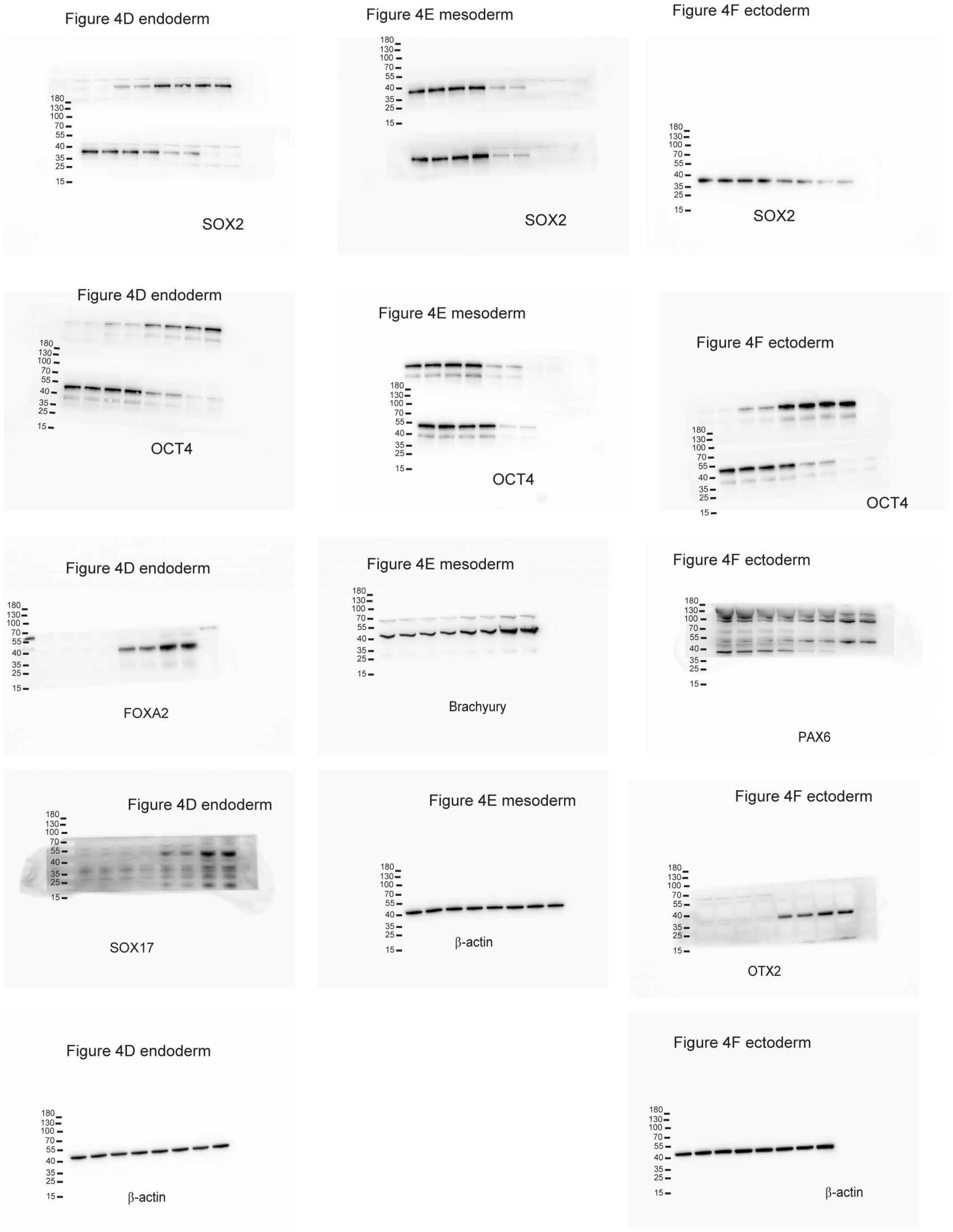

Supplementary Figure. 18

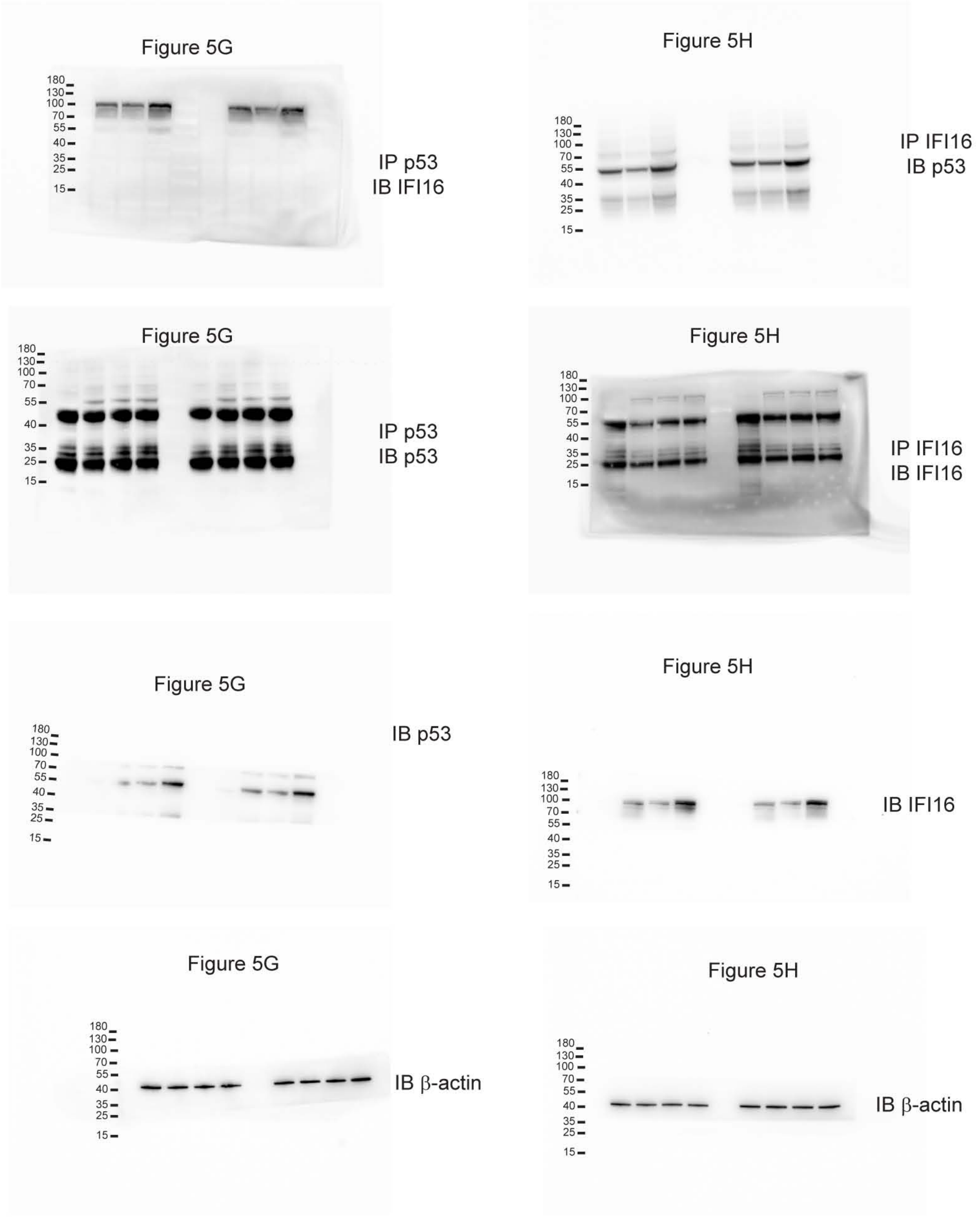

Supplementary Figure. 19

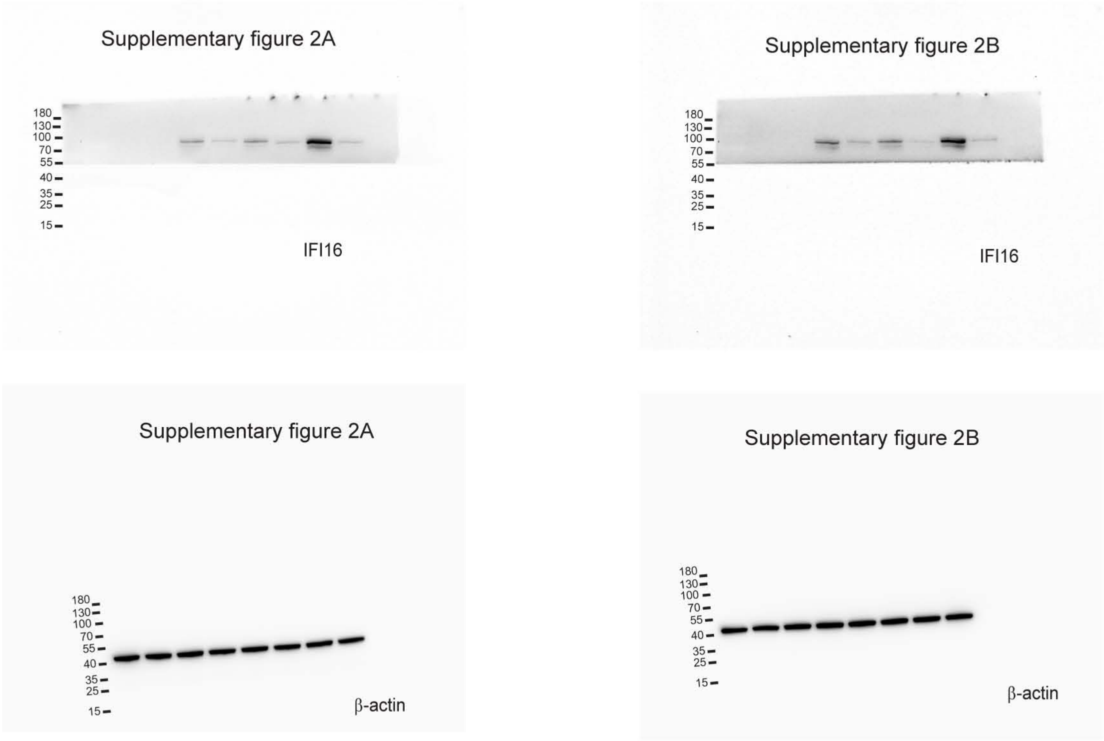

Supplementary Figure. 20

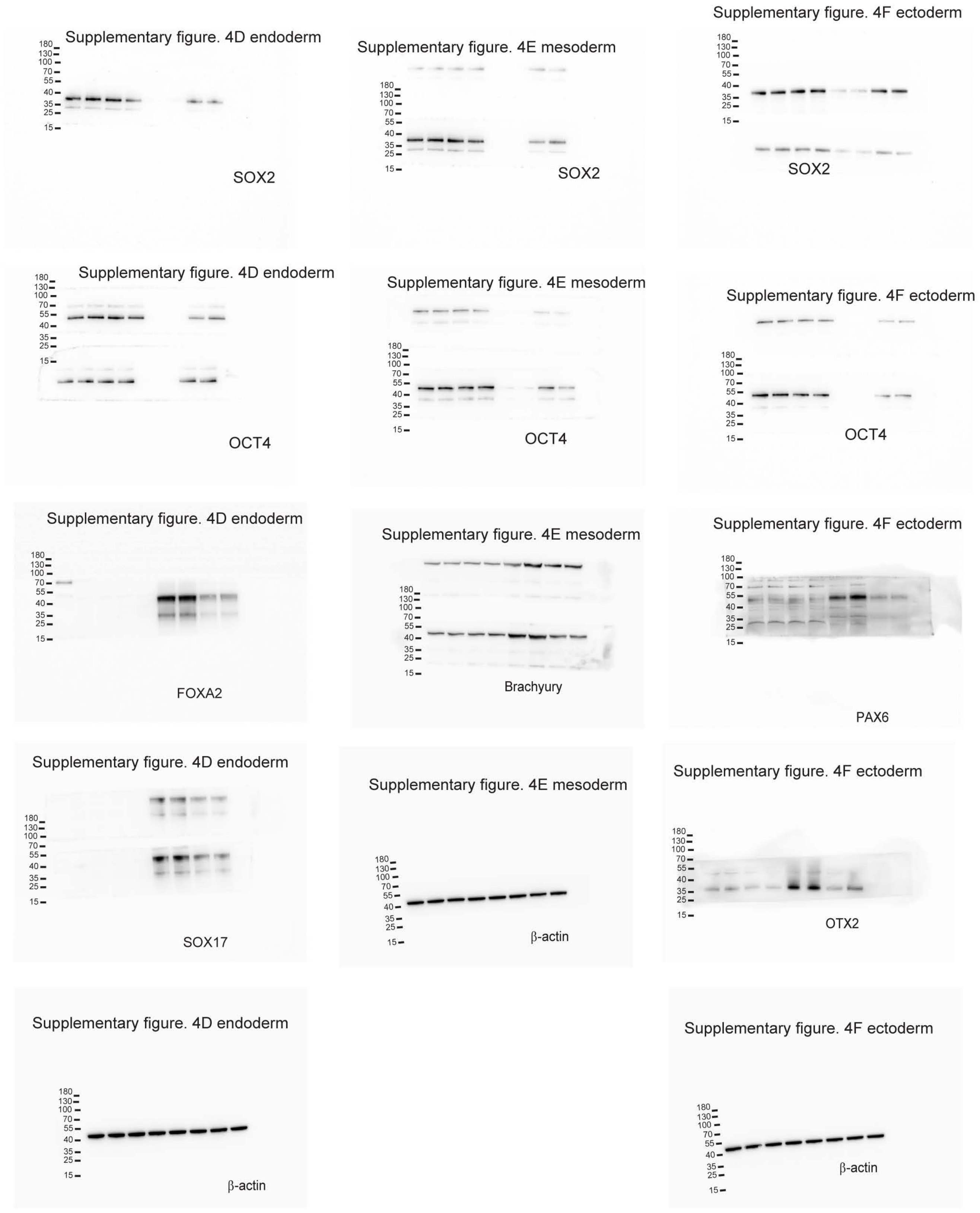

Supplementary Figure. 21

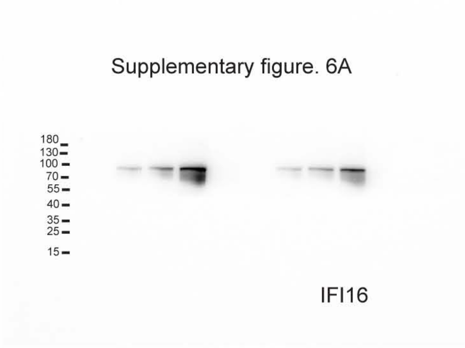

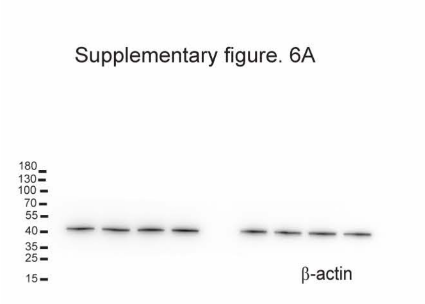

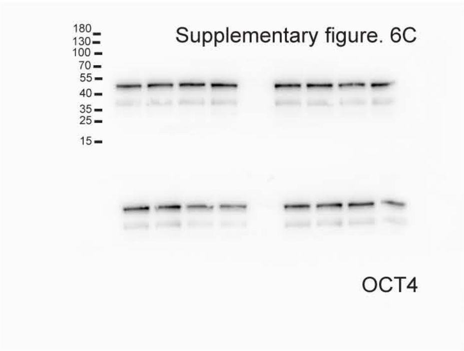

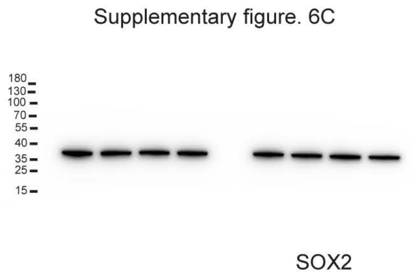

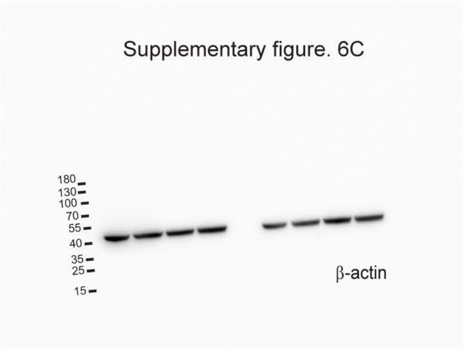

Supplementary Figure. 22

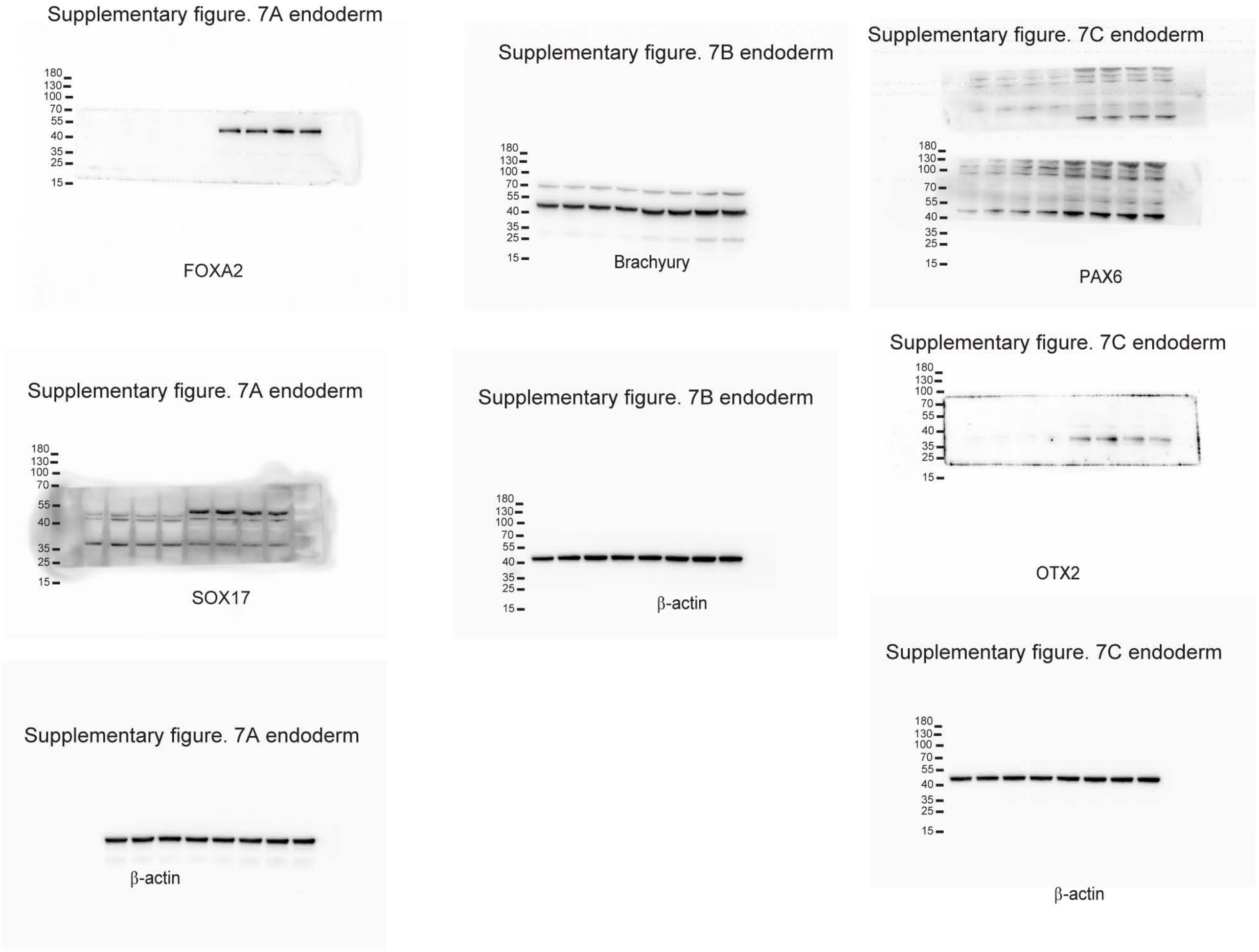

Supplementary Figure. 23

Supplementary figure legends

Supplementary Figure. 1. Expression levels of IFI16, AIM2, and cGAS during trilineage specification.

a-c, quantitative PCR analysis of AIM2 and cGAS mRNA levels in H9 cells and differentiated trilineage (a, endoderm; b, mesoderm; c, ectoderm; n=4 in each group). d-f, statistics for immunoblots of IFI16 and AIM2 from H9 cells and differentiated trilineage (d, endoderm; e, mesoderm; f, ectoderm) for indicated periods of time respectively, n=8 for IFI16 and n=4 for AIM2 in each group. g-i, statistics for immunoblots of cGAS from H9 cells and differentiated trilineage (g, endoderm, n=6; h, mesoderm, n=4; i, ectoderm, n=4) for indicated periods of time. The relative mRNA and protein levels of genes in indicated time courses were calculated relatively to which in H9 cells. Comparisons between groups for statistical significance were performed with one-way ANOVA with Tukey's post hoc test. **P＜0.01, ***P＜0.001 versus H9.

Supplementary Figure. 2. IFI16 knockdown by sh1865 and sh2153 trilineage specification.

a-b, western blot analysis of expression of IFI16 in H9 cells and differentiated trilineage infected with sh1865 (a) and sh2153 (b), shNC is used as a negative control (NC) and β-actin serves as a loading control. c-d, statistics results for A-C respectively (C, sh1865; D, sh2153; n=8 in each group). The relative protein level of IFI16 was calculated relatively to which in H9-shNC group. e-h, CCK-8 assay analysis of the cell viability in H9 cells and differentiated trilineage (e, H9 n=8; f, endoderm n=4; g, mesoderm n=4; h, ectoderm n=4) after IFI16 knockdown. The relative absorbance value was calculated relatively to which in H9-shNC or Day1-shNC group. Comparisons between groups for statistical significance were performed with two-way ANOVA with Bonferroni post hoc test (c-d and f-h), or Student’s t test, two tails (E). ***P＜0.001 versus shNC.

Supplementary Figure. 3. sh1865 inhibits trilineage specification.

a-c, statistics for immunoblots of OCT4, SOX2, SOX17, FOXA2, Brachyury, PAX6, and OTX2 expression in H9 cells and differentiated trilineage (a, endoderm; b, mesoderm; c, ectoderm; n=8 in each group) infected with shNC and sh1865. The relative protein level of each gene was calculated relatively to which in H9-shNC group. d-f, statistic analysis for the proportion of SOX17^{+}/FOXA2^{+} (d), Brachyury^{+}/CXCR4^{+} (e), and PAX6+/Nestin^{+} (f) population in different batches of differentiated trilineage infected with shNC or sh1865 (n=3 in each group). The relative doubly-positive cell rate was calculated relatively to which in shNC group. g-i, statistics for immunofluorescence analysis of the expression of OCT4 (n=8), SOX2 (n=8), SOX17 (n=8), FOXA2 (n=8), Brachyury (n=7), SNAI2 (n=7), and PAX6 (n=7) in the differentiated trilineage infected with sh1865 or shNC. AOD, average optical density. The relative AOD of immunoreactivity was calculated relatively to which in shNC group. ENDO, endoderm; MESO, mesoderm; ECTO, ectoderm. NC, negative control. All data were presented as mean ± SEM. Comparisons between groups for statistical significance were performed with one-way ANOVA with Tukey's post hoc test (a-c), Student’s t test, two tails (d-f), or two-way ANOVA with Bonferroni post hoc test (g-i). *P＜0.05, **P＜0.01, ***P＜0.001 versus H9-NC or shNC. #P＜0.05, ###P＜0.001 versus H9-ENDO, H9-MESO, or H9-ECTO.

Supplementary Figure. 4. IFI16 knockdown by sh2153 inhibits trilineage specification.

a-c, quantitative PCR examination of OCT4, SOX2, SOX17, FOXA2, CXCR4, Brachyury, PAX6, and OTX2 mRNA levels in H9 cells and differentiated trilineage (a, endoderm; b, mesoderm; c, ectoderm; n=4 in each group) infected with sh2153 or shNC. The relative mRNA level of each gene was calculated relatively to which in H9-shNC group. d-f, representative immunoblots of total lysates from H9 cells and differentiated trilineage (d, endoderm; e, mesoderm; f, ectoderm) infected with sh2153 or shNC and probed with the antibodies for OCT4, SOX2, SOX17, FOXA2, Brachyury, PAX6, and OTX2. β-actin serves as a loading control. g, flow cytometric analysis of SOX17^{+}/FOXA2^{+}, Brachyury^{+}/CXCR4^{+}, and PAX6^{+}/Nestin^{+} population

in differentiated trilineage infected with sh2153 or shNC, The signals in the fourth quadrant indicate endoderm, mesoderm, or ectoderm population. The number in each quadrant means the proportion in total cell population. h, representative immunofluorescence images staining with antibodies against OCT4, SOX2, SOX17, FOXA2, Brachyury, SNAI2, and PAX6 in the differentiated trilineage infected with sh2153 or shNC. Upper rows, endoderm; middle rows, mesoderm; bottom rows, ectoderm. DAPI serves as a nucleus indicator. Scale bar, 200 μM. ENDO, endoderm; MESO, mesoderm; ECTO, ectoderm. NC, negative control. All data were presented as mean ± SEM. Comparisons between groups for statistical significance were performed with one-way ANOVA with Tukey's post hoc test (a-c). *P＜0.05, **P＜0.01, ***P＜0.001 versus H9-NC. #P＜0.05, ##P＜0.01, ###P＜0.001 versus H9-ENDO, H9-MESO, or H9-ECTO.

Supplementary Figure. 5. sh2153 inhibits trilineage specification.

a-c, statistics results for immunoblots of OCT4, SOX2, SOX17, FOXA2, Brachyury, PAX6, and OTX2 expression in H9 cells and differentiated trilineage (a, endoderm; b, mesoderm; c, ectoderm; n=4 in each group) infected with shNC and sh2153. The relative protein level of each gene was calculated relatively to which in H9-shNC group. d-f, statistic analysis for the proportion of SOX17$^+$/FOXA2$^+$ (d), Brachyury$^+$/CXCR4$^+$ (e), and PAX6$^+$/Nestin$^+$ (f) population in different batches of differentiated trilineage infected with shNC or sh2153 (n=3 in each group). The relative doubly-positive cell rate was calculated relatively to which in shNC group. g-i, statistics for immunofluorescence analysis of the expression of OCT4 (n=6), SOX2 (n=6), SOX17 (n=5), FOXA2 (n=5), Brachyury (n=5), SNAI2 (n=5), and PAX6 (n=5) in the differentiated trilineage infected with sh2153 or shNC. AOD, average optical density. The relative AOD of immunoreactivity was calculated relatively to which in shNC group. ENDO, endoderm; MESO, mesoderm; ECTO, ectoderm. NC, negative control.All data were presented as mean ± SEM. Comparisons between groups for statistical significance were performed with one-way ANOVA with Tukey's post hoc test (a-c), Student’s t test, two tails (d-f) or two-way ANOVA with Bonferroni post

hoc test (g-i). *P＜0.05, **P＜0.01, ***P＜0.001 versus H9-NC or shNC. #P＜0.05, ##P＜0.01, ###P＜0.001 versus H9-ENDO, H9-MESO, or H9-ECTO.

Supplementary Figure. 6. IFI16 overexpression alone does not affect proliferation and self-renewal gene expression in hESCs.

a-b, western blot analysis of DOX-induced IFI16 expression in H9 cells infected with IFI16 and NC (n=8 in each group). The relative protein level of IFI16 was calculated relatively to which in D0 group. c, alkaline phosphatase staining of DOX-induced IFI16 expressed H9 cells infected with IFI16 and NC. d-e, western blot analysis of OCT4 and SOX2 expression of DOX-induced IFI16 expressed H9 cells infected with IFI16 and NC (n=4 for OCT4 and n=4 for SOX2). β-actin serves as a loading control. The relative protein level was calculated relatively to which in H9-NC DOX^- group. DOX: Doxycycline. NC, negative control. All data were presented as mean ± SEM. Comparisons between groups for statistical significance were performed with one-way ANOVA with Tukey's post hoc test (b) or two-way ANOVA with Bonferroni post hoc test (e). *P＜0.05, ***P＜0.001 versus D0, or H9-NC DOX^-.

Supplementary Figure. 7. IFI16 overexpression does not affect differentiated gene expression in the late stages of trilineage induction.

a-c, representative immunoblots of total lysates from H9 cells and differentiated trilineage on Day 5 or Day 7 (a, endoderm; b, mesoderm; c, ectoderm) infected with IFI16 or NC and probed with the antibodies for OCT4, SOX2, SOX17, FOXA2, Brachyury, PAX6, and OTX2. β-actin serves as a loading control. The relative protein level of each gene was calculated relatively to which in H9-NC group. d-f, statistics results for a-c respectively, n=4 for each group. g-i, flow cytometric analysis of $SOX17^+$/$FOXA2^+$, $Brachyury^+$/$CXCR4^+$, and $PAX6^+$/$Nestin^+$ population in differentiated trilineage on Day 5 or Day 7 infected with IFI16 or NC (n=3 for each group). The signals in the fourth quadrant indicate endoderm, mesoderm, or ectoderm population. The number in each quadrant means the proportion in total cell population. The right histograms are statistic results for the proportion of $SOX17^+$/$FOXA2^+$,

Brachyury$^+$/CXCR4$^+$, and PAX6+/Nestin$^+$ population in different batches. The relative doubly-positive cell rate was calculated relatively to which in NC group. ENDO, endoderm; MESO, mesoderm; ECTO, ectoderm. NC, negative control. All data were presented as mean ± SEM. Comparisons between groups for statistical significance were performed with one-way ANOVA with Tukey's post hoc test (d-f) or Student’s t test, two tails (g-i). **P＜0.01, ***P＜0.001 versus H9-NC.

Supplementary Figure. 8. DOX induced IFI16 expression accelerates trilineage specification.

a-c, statistics results for immunoblots of OCT4, SOX2, SOX17, FOXA2, Brachyury, PAX6, and OTX2 expression in H9 cells and differentiated trilineage on Day 2 (a, endoderm; b, mesoderm; c, ectoderm; n=4 for OCT4, SOX2, and Brachyury; n=6 for SOX17, FOXA2, PAX6, and OTX2) infected with IFI16 or NC. The relative protein level of each gene was calculated relatively to which in H9-NC group. d-f, statistic analysis for the proportion of SOX17$^+$/FOXA2$^+$ (d), Brachyury$^+$/CXCR4$^+$ (e), and PAX6+/Nestin$^+$ (f) population in different batches of differentiated trilineage infected with NC or IFI16 (n=3 in each group). The relative doubly-positive cell rate was calculated relatively to which in NC group. g-i, statistics for immunofluorescence analysis of the expression of OCT4 (n=8), SOX2 (n=8), SOX17 (n=7), FOXA2 (n=7), Brachyury (n=7), SNAI2 (n=7), and PAX6 (n=7) in the differentiated trilineage infected with IFI16 or NC. AOD, average optical density. The relative AOD of immunoreactivity was calculated relatively to which in shNC group. ENDO, endoderm; MESO, mesoderm; ECTO, ectoderm. NC, negative control. All data were presented as mean ± SEM. Comparisons between groups for statistical significance were performed with one-way ANOVA with Tukey's post hoc test (a-c), Student’s t test, two tails (d-f) or two-way ANOVA with Bonferroni post hoc test (g-i). *P＜0.05, **P＜0.01, ***P＜0.001 versus H9-NC or NC. #P＜0.05, ##P＜0.01, ###P＜0.001 versus H9-ENDO, H9-MESO, or H9-ECTO.

Supplementary Figure. 9. Motif and gene ontology analysis of p53 peaks during

trilineage specification in shNC group.

a, enriched p53 binding motifs in differentiated trilineage. b-d, biological processes gene ontology (GO) analysis for genes enriched by p53-shNC peaks in differentiated trilineage (b, endoderm; c, mesoderm; d, ectoderm). Each node represented a specific GO term, and the node size indicated the number of genes in the GO term. The GO term annotations were listed in the Supplementary Dataset 1. ENDO, endoderm; MESO, mesoderm; ECTO, ectoderm.

Supplementary Figure. 10. Gene ontology analysis of differentiated expressed genes during trilineage specification after IFI16 knockdown.

a-c, biological processes gene ontology (GO) analysis for genes differentially expressed after IFI16 knockdown in differentiated trilineage (a, endoderm; b, mesoderm; c, ectoderm). Each node represented a specific GO term, and the node size indicated the number of genes in the GO term. The GO term annotations were listed in the Supplementary Dataset 1. ENDO, endoderm; MESO, mesoderm; ECTO, ectoderm. d, volcano maps of p53-shNC special peak enriched genes and genes differentially expressed after IFI16 knockdown during trilineage specification (left, endoderm; middle, mesoderm; right, ectoderm). The numbers in each volcano map indicate the amount of p53-activated (left) and p53-repressed (right) genes.

Supplementary Figure. 11. The isotype-mouse IgG1/rabbit IgG immunofluorescence staining in H9 cells and the differentiated trilineage.

The immunofluorescence images staining with mouse IgG1/rabbit IgG as the isotype control in H9 cells and the differentiated trilineage. DAPI serves as a nucleus indicator. 488 or 594 indicated the Alexa Fluor 488/594 conjugated secondary antibody. Scale bar, 200 µM.

Supplementary Figure. 12. The isotype-mouse IgG1/rabbit IgG immunofluorescence staining in H9 cells and the differentiated trilineage after IFI16 knockdown.

The immunofluorescence images staining with mouse IgG1/rabbit IgG as the isotype control

in H9 cells and the differentiated trilineage infected with shNC, sh1865 or sh2153. DAPI serves as a nucleus indicator. 488 or 594 indicated the Alexa Fluor 488/594 conjugated secondary antibody. Scale bar, 200 μM.

Supplementary Figure. 13. The isotype-mouse IgG1/rabbit IgG immunofluorescence staining in H9 cells and the differentiated trilineage after IFI16 overexpression.

The immunofluorescence images staining with mouse IgG1/rabbit IgG as the isotype control in H9 cells and the differentiated trilineage infected with NC or IFI16. DAPI serves as a nucleus indicator. 488 or 594 indicated the Alexa Fluor 488/594 conjugated secondary antibody. Scale bar, 200 μM.

Supplementary Figure. 14. The flow cytometric analysis of isotype staining in the differentiated trilineage.

Representative flow cytometric plots of isotype staining in the differentiated trilineage infected with shNC/sh1865/sh2153 or NC/IFI16. ENDO, endoderm; MESO, mesoderm; ECTO, ectoderm.

Supplementary Figure. 15. The the uncropped blots for Figure 1.

Supplementary Figure. 16. The the uncropped blots for Figure 2.

Supplementary Figure. 17. The the uncropped blots for Figure 3.

Supplementary Figure. 18. The the uncropped blots for Figure 4.

Supplementary Figure. 19. The the uncropped blots for Figure 5.

Supplementary Figure. 20. The the uncropped blots for Supplementary Figure 2.

Supplementary Figure. 21. The the uncropped blots for Supplementary Figure 4.

Supplementary Figure. 22. The the uncropped blots for Supplementary Figure 6.

Supplementary Figure. 23. The the uncropped blots for Supplementary Figure 7.

Supplementary Table 1.Plasmids, shRNAs, and primers

qPCR primers	
GAPDH	Forward ACATCATCCCTGCCTCTACTG<br>Reverse ACCACCTGGTGCTCAGTGTA
IFI16	Forward TTGATTAGAAGTGCCAGCGTA<br>Reverse GATTGTGGTCAGTCGTCCATG
IFI16 ChIP promoter	Forward CTCCTCCTGCCACTGCCACTCTG<br>Reverse CTTACCCAGCAGACTTACATGTGAGTC
IFI16 ChIP 3’UTR	Forward GTTTGCCGCAATGGGTTCC<br>Reverse ATCTCCATGTTTCGGTCAGCA
AIM2 ChIP promoter	Forward CACAGAAGAAACTGGCCGCAACTG<br>Reverse TCATGGCGTCAGGTGCATGATGG
cGAS ChIP promoter	Forward CTCTAGCCCCTGGCAACCACCA<br>Reverse CTTGCAACATAGATGAACATTGGCT
AIM2	Forward TGGCAAAACGTCTTCAGGAGG<br>Reverse AGCTTGACTTAGTGGCTTTGG
cGAS	Forward TAACCCTGGCTTTGGAATCAAAA<br>Reverse TGGGTACAAGGTAAAATGGCTTT
OCT4	Forward GGGGTTCTATTTGGGAAGGTAT<br>Reverse TACTGGTTCGCTTTCTCTTTCG
SOX2	Forward GCTCGCAGACCTACATGAAC<br>Reverse GGGAGGAAGAGGTAACCACA
Nanog	Forward ATAACCTTGGCTGCCGTCTC<br>Reverse AGCCTCCCAATCCCAAACAA
KLF4	Forward ATTACGCGGGCTGCGGCAAAA<br>Reverse TTTTTGGCACTGGAACGGGCGG
SOX17	Forward AAGGGCGAGTCCCGTATC<br>Reverse GTACTTGTAGTTGGGGTGGTCCT
FOXA2	Forward CAAGGGCCAGAGTTCCACAA<br>Reverse CCTGCAACCAGACAGGGTAT
CXCR4	Forward CTCCTCTTTGTCATCACGCTTCC<br>Reverse GGATGAGGACACTGCTGTAGAG
Brachyury	Forward CTATTCTGACAACTCACCTGCAT<br>Reverse ACAGGCTGGGGTACTGACT
PAX6	Forward TGGGCAGGTATTACGAGACTG<br>Reverse ACTCCCGCTTATACTGGGCTA
OTX2	Forward CATGCAGAGGTCCTATCCCAT<br>Reverse AAGCTGGGGACTGATTGAGAT
shRNA sequences	
IFI16 sh1865	GGTGCTGAACGCAACAGAA
IFI16 sh2153	GCTTTGCTCACAAACTAAA

IFI16 sequence

ATGGGAAAAAAATACAAGAACATTGTTCTACTAAAAGGATTAGAGGTCATCAATGA
TTATCATTTTAGAATGGTTAAGTCCTTACTGAGCAACGATTTAAAACTTAATTTAAA
AATGAGAGAAGAGTATGACAAAATTCAGATTGCTGACTTGATGGAAGAAAAGTTC
CGAGGTGATGCTGGTTTGGGCAAACTAATAAAAATTTTCGAAGATATACCAACGCT
TGAAGACCTGGCTGAAACTCTTAAAAAAGAAAAGTTAAAAGTAAAAGGACCAGC
CCTATCAAGAAAGAGGAAGAAGGAAGTGGATGCTACTTCACCTGCACCCTCCACA
AGCAGCACTGTCAAAACTGAAGGAGCAGAGGCAACTCCTGGAGCTCAGAACCCG
AAAACAGTGGCCAAATGTCAGGTAACTCCCAGAAGAAATGTTCTCCAAAAACGCC
CAGTGATAGTGAAGGTACTGAGTACAACAAAGCCATTTGAATATGAGACCCCAGA
AATGGAGAAAAAAATAATGTTTCATGCTACAGTGGCTACACAGACACAGTTCTTCC
ATGTGAAGGTTTTAAACACCAGCTTGAAGGAGAAATTCAATGGAAAGAAAATCAT
CATCATATCAGATTATTTGGAATATGATAGTCTCCTAGAGGTCAATGAAGAATCTACT
GTATCTGAAGCTGGTCCTAACCAAACGTTTGAGGTTCCAAATAAAATCATCAACAG
AGCAAAGGAAACTCTGAAGATTGATATTCTTCACAAACAAGCTTCAGGAAATATTG
TATATGGGGTATTTATGCTACATAAGAAAACAGTAAATCAGAAGACCACAATCTACG
AAATTCAGGATGATAGAGGAAAAATGGATGTAGTGGGGACAGGACAATGTCACAA
TATCCCCTGTGAAGAAGGAGATAAGCTCCAACTTTTCTGCTTTCGACTTAGAAAAA
AGAACCAGATGTCAAAACTGATTTCAGAAATGCATAGTTTTATCCAGATAAAGAAA
AAAACAAACCCGAGAAACAATGACCCCAAGAGCATGAAGCTACCCCAGGAACAG
CGTCAGCTTCCATATCCTTCAGAGGCCAGCACAACCTTCCCTGAGAGCCATCTTCG
GACTCCTCAGATGCCACCAACAACTCCATCCAGCAGTTTCTTCACCAAGAAAAGT
GAAGACACAATCTCCAAAATGAATGACTTCATGAGGATGCAGATACTGAAGGAAG
GGAGTCATTTTCCAGGACCGTTCATGACCAGCATAGGCCCAGCTGAGAGCCATCCC
CACACTCCTCAGATGCCTCCATCAACACCAAGCAGCAGTTTCTTAACCACGAAAA
GTGAAGACACAATCTCCAAAATGAATGACTTCATGAGGATGCAGATACTGAAGGA
AGGGAGTCATTTTCCAGGACCGTTCATGACCAGCATAGGCCCAGCTGAGAGCCAT
CCCCACACTCCTCAGATGCCTCCATCAACACCAAGCAGCAGTTTCTTAACCACGTT
GAAACCAAGACTGAAGACTGAACCTGAAGAAGTTTCCATAGAAGACAGTGCCCA
GAGTGACCTCAAAGAAGTGATGGTGCTGAACGCAACAGAATCATTTGTATATGAG
CCCAAAGAGCAGAAGAAAATGTTTCATGCCACAGTGGCAACTGAGAATGAAGTCT
TCCGAGTGAAGGTTTTTAATATTGACCTAAAGGAGAAGTTCACCCCAAAGAAGAT
CATTGCCATAGCAAATTATGTTTGCCGCAATGGGTTCCTGGAGGTATATCCTTTCAC
ACTTGTGGCTGATGTGAATGCTGACCGAAACATGGAGATCCCAAAAGGATTGATTA
GAAGTGCCAGCGTAACTCCTAAAATCAATCAGCTTTGCTCACAAACTAAAGGAAG
TTTTGTGAATGGGGTGTTTGAGGTACATAAGAAAAATGTAAGGGGTGAATTCACTT
ATTATGAAATACAAGATAATACAGGGAAGATGGAAGTGGTGGTGCATGGACGACTG
ACCACAATCAACTGTGAGGAAGGAGATAAACTGAAACTCACCTGCTTTGAATTGG
CACCGAAAAGTGGGAATACCGGGGAGTTGAGATCTGTAATTCATAGTCACATCAA
GGTCATCAAGACCAGGAAAAACAAGAAAGACATACTCAATCCTGATTCAAGTATG
GAAACTTCACCAGACTTTTTCTTC

Supplementary Data legend

The GO term annotations for Figure 6e-g, Supplementary Figure. 9b-d, and Supplementary Figure. 10a-c.
