# Supplementary material for: IFI16 promotes human embryonic stem cell trilineage specification through interaction with p53
Source: NPJ Regen Med. 2020 Oct 29;5:18. doi: 10.1038/s41536-020-00104-0 (PMC7596047; doi:10.1038/s41536-020-00104-0)
Supplement: Supplementary file 3 — Reporting Summary [file 41536_2020_104_MOESM3_ESM.pdf]

nature research

Corresponding author(s): Furong Li, Ning Li, Xiaofei Yang
NPJREGENMED-00372

Last updated by author(s): Jul 12, 2020

Reporting Summary

Nature Research wishes to improve the reproducibility of the work that we publish. This form provides structure for consistency and transparency in reporting. For further information on Nature Research policies, see our Editorial Policies and the Editorial Policy Checklist.

Statistics

For all statistical analyses, confirm that the following items are present in the figure legend, table legend, main text, or Methods section.

n/a	Confirmed	
☐	☒	The exact sample size (*n*) for each experimental group/condition, given as a discrete number and unit of measurement
☐	☒	A statement on whether measurements were taken from distinct samples or whether the same sample was measured repeatedly
☐	☒	The statistical test(s) used AND whether they are one- or two-sided<br>*Only common tests should be described solely by name; describe more complex techniques in the Methods section.*
☒	☐	A description of all covariates tested
☒	☐	A description of any assumptions or corrections, such as tests of normality and adjustment for multiple comparisons
☐	☒	A full description of the statistical parameters including central tendency (e.g. means) or other basic estimates (e.g. regression coefficient) AND variation (e.g. standard deviation) or associated estimates of uncertainty (e.g. confidence intervals)
☐	☒	For null hypothesis testing, the test statistic (e.g. *F*, *t*, *r*) with confidence intervals, effect sizes, degrees of freedom and *P* value noted<br>*Give P values as exact values whenever suitable.*
☒	☐	For Bayesian analysis, information on the choice of priors and Markov chain Monte Carlo settings
☒	☐	For hierarchical and complex designs, identification of the appropriate level for tests and full reporting of outcomes
☒	☐	Estimates of effect sizes (e.g. Cohen's *d*, Pearson's *r*), indicating how they were calculated

Our web collection on statistics for biologists contains articles on many of the points above.

Software and code

Policy information about availability of computer code

Data collection	N/A
Data analysis	N/A

For manuscripts utilizing custom algorithms or software that are central to the research but not yet described in published literature, software must be made available to editors and reviewers. We strongly encourage code deposition in a community repository (e.g. GitHub). See the Nature Research guidelines for submitting code & software for further information.

Data

Policy information about availability of data

All manuscripts must include a data availability statement. This statement should provide the following information, where applicable:

- Accession codes, unique identifiers, or web links for publicly available datasets
- A list of figures that have associated raw data
- A description of any restrictions on data availability

ChIP-seq and RNA-seq raw data and processing data supporting this work have been upload to website GEO under the accession number GSE142050.

Field-specific reporting

Please select the one below that is the best fit for your research. If you are not sure, read the appropriate sections before making your selection.

☒ Life sciences ☐ Behavioural & social sciences ☐ Ecological, evolutionary & environmental sciences

For a reference copy of the document with all sections, see nature.com/documents/nr-reporting-summary-flat.pdf

Life sciences study design

All studies must disclose on these points even when the disclosure is negative.

Sample size	No sample size calculations were performed. Three or more biological replicates were carried out for each experiment.
Data exclusions	N/A
Replication	Replication was successful.
Randomization	N/A
Blinding	N/A

Reporting for specific materials, systems and methods

We require information from authors about some types of materials, experimental systems and methods used in many studies. Here, indicate whether each material, system or method listed is relevant to your study. If you are not sure if a list item applies to your research, read the appropriate section before selecting a response.

Materials & experimental systems

n/a	Involved in the study	
☐	☒	Antibodies
☐	☒	Eukaryotic cell lines
☒	☐	Palaeontology and archaeology
☒	☐	Animals and other organisms
☒	☐	Human research participants
☒	☐	Clinical data
☒	☐	Dual use research of concern

Methods

n/a	Involved in the study	
☐	☒	ChIP-seq
☐	☒	Flow cytometry
☒	☐	MRI-based neuroimaging

Antibodies

Antibodies used	IFI16 from Abcam (ab169788, Western blots/Co-IP) and Santa Cruz (sc-8023, PLA/Immunofluorescence), p53 from CST (2527, PLA) and Santa Cruz (sc-126, ChIP/ Western blots/Co-IP), AIM2 from Abcam (ab180665, Western blots/ Immunofluorescence), OCT4 from Abcam (ab19857, Western blots/ Immunofluorescence), SOX2 from CST (2748S, Western blots/ Immunofluorescence), Brachyury from Abcam (ab20680, Western blots/ Immunofluorescence), OTX2 from Abcam (ab21990, Western blots/ Immunofluorescence), PAX6 from Abcam (ab5790, Western blots/ Immunofluorescence), SOX17 from Abcam (ab84990, Western blots/ Immunofluorescence), SNAI2 from Santa Cruz (sc-166476, Immunofluorescence), FOXA2 from R&D (AF2400, Western blots/ Immunofluorescence), c-Jun from Abcam (ab31419, Western blots), p38 from CST (9212s, Western blots), p-p38 from CST (9211s, Western blots), ERK from CST (4695s, Western blots), p-ERK from CST (4370s, Western blots), JNK from CST (9252s, Western blots), p-JNK from Santa Cruz (sc-6254, Western blots), CXCR4-FITC from R&D (FAB170F, Flow cytometry), Brachyruy-APC from R&D (IC2085A, Flow cytometry), SOX17-APC from R&D (IC1924A, Flow cytometry), FOXA2-488 from R&D (IC2400G, Flow cytometry), Nestin-APC from R&D (IC1259V, Flow cytometry), PAX6-488 from BD (561664, Flow cytometry), and β-actin from ZEN BIO (200058-BF10, Western blots).
Validation	For validation, the isotypes were introduced as negative controls to test the specificity of primary antibodies.

Eukaryotic cell lines

Policy information about cell lines

Cell line source(s)	Human embryonic stem cell (hESC) line H9 (order number: 18-1-1522) was from Cell Bank of the Shanghai Institutes for Biological Sciences of the Chinese Academy of Sciences.
Authentication	H9 cells were authenticated using Short Tandem Repeat (STR) analysis (GENETIC TESTING BIOTECHNOLOGY Co., Ltd.).

Mycoplasma contamination	H9 cells were tested negative for mycoplasma.
Commonly misidentified lines (See ICLAC register)	N/A

ChIP-seq

Data deposition

☒ Confirm that both raw and final processed data have been deposited in a public database such as GEO.

☒ Confirm that you have deposited or provided access to graph files (e.g. BED files) for the called peaks.

Data access links *May remain private before publication.*	ChIP-seq raw data and processing data supporting this work have been upload to website GEO under the accession number GSE142050.
Files in database submission	GSM4218343 ENDONC_1<br>GSM4218344 ENDONC_2<br>GSM4218345 ENDO1865_1<br>GSM4218346 ENDO1865_2<br>GSM4218347 MESONC_1<br>GSM4218348 MESONC_2<br>GSM4218349 MESO1865_1<br>GSM4218350 MESO1865_2<br>GSM4218351 ECTONC_1<br>GSM4218352 ECTONC_2<br>GSM4218353 ECTO1865_1<br>GSM4218354 ECTO1865_2<br>GSM4218355 ENDOINPUT<br>GSM4218356 MESOINPUT<br>GSM4218357 ECTOINPUT
Genome browser session (e.g. UCSC)	N/A

Methodology

Replicates	two replicates for each sample
Sequencing depth	The libraries were sequencing with PE150, the raw reads number was between 80-120 million, and the unique mapped ratio was between 84-88%.
Antibodies	p53 from Santa Cruz (sc-126)
Peak calling parameters	bowtie2 -x Genome.fa -S sample.sam -N(allowing at most one mismacht) 1 -1 -2 and macs2 -t sample.bam -c input.bam -f BAM -g genome_size -n sample_name -p 1e-5 for peak calling
Data quality	Filtering low quality (mapQ>=30) reads, and for bam files: p-value <1e-5 , FDR <= 0.05, peak enrichment >20, peak-to-background enrichment >3, and a kernel bandwidth of 300.
Software	Softwares for data analysis were indicated in the Materials and Methods.

Flow Cytometry

Plots

Confirm that:

☒ The axis labels state the marker and fluorochrome used (e.g. CD4-FITC).

☒ The axis scales are clearly visible. Include numbers along axes only for bottom left plot of group (a 'group' is an analysis of identical markers).

☒ All plots are contour plots with outliers or pseudocolor plots.

☒ A numerical value for number of cells or percentage (with statistics) is provided.

Methodology

Sample preparation	Briefly, the cultured cells were dissociated into single cells and resuspended in 0.1 mM PBS. After centrifuging at 300 g for 5 minutes, cells were incubated with fixation/permeabilization solution for 20 miniutes at room temperature. Sequentially, the diluted BD Perm/Wash™ Buffer was used to wash the cells twice. Then antibodies of marker protein (endoderm: SOX17-APC, FOXA2-488; mesoderm: Brachyruy-APC, CXCR4-FITC; ectoderm: Nestin-APC, PAX6-488) and isotypes were then added for staining.

Instrument	Beckman DxFLEX
Software	FlowJo_V10
Cell population abundance	Isotypes were introduced as negative control to eliminate the non-specific staining.
Gating strategy	Gating was based on antibodies and isotypes (1 ug for 1 million cells)that were added to the cells.

☒ Tick this box to confirm that a figure exemplifying the gating strategy is provided in the Supplementary Information.
